# Synthesis, In Silico and In Vitro Characterization of Novel *N*,*N*-Substituted Pyrazolopyrimidine Acetamide Derivatives for the 18KDa Translocator Protein (TSPO)

**DOI:** 10.3390/ph16040576

**Published:** 2023-04-11

**Authors:** Jaekyung Park, Sobia Wasim, Jae Ho Jung, Mi-hyun Kim, Byung Chul Lee, Mohammad Maqusood Alam, Sang-Yoon Lee

**Affiliations:** 1 Gachon Advanced Institute for Health Science and Technology, Graduate School, Gachon University, Incheon 21999, Republic of Korea; 2 Department of Biochemistry, College of Medicine, Gachon University, Incheon 21999, Republic of Korea; 3 Department of Nuclear Medicine, Seoul National University College of Medicine, Seoul National University Bundang Hospital, Seongnam 13620, Republic of Korea; 4 Gachon Institute of Pharmaceutical Science and Department of Pharmacy, College of Pharmacy, Gachon University, Incheon 21936, Republic of Korea; 5 Center for Nanomolecular Imaging and Innovative Drug Development, Advanced Institutes of Convergence Technology, Suwon 16229, Republic of Korea; 6 Neuroscience Research Institute, Gachon University, Incheon 20565, Republic of Korea; 7 Department of Neuroscience, College of Medicine, Gachon University, Incheon 21936, Republic of Korea

**Keywords:** translocator protein (TSPO), microglia, neuroinflammation, neurodegenerative disease, central nervous system (CNS), pyrazolopyrimidine, molecular docking, MD simulation

## Abstract

The translocator protein (TSPO) is an interesting biological target for molecular imaging and therapy because the overexpression of TSPO is associated with microglial activation caused by neuronal damage or neuroinflammation, and these activated microglia are involved in various central nervous system (CNS) diseases. The TSPO is a target for neuroprotective treatment, which is used with the aim of reducing microglial cell activation. The novel *N*,*N*-disubstituted pyrazolopyrimidine acetamides scaffold (**GMA 7**–**17**), which bears a fluorine atom and is directly linked to the phenyl moiety, was synthesized, and each of the novel ligands was characterized in vitro. All of the newly synthesized ligands displayed picomolar to nanomolar affinity for the TSPO. Particularly, an in vitro affinity study led to the discovery of 2-(5,7-diethyl-2-(4-fluorophenyl)pyrazolo [1,5-a]pyrimidin-3-yl)-*N*-ethyl-*N*-phenylacetamide **GMA 15** (*Ki* = 60 pM), a novel TSPO ligand that exhibits a 61-fold enhancement in affinity compared to the reference standard DPA-714 (*Ki* = 3.66 nM). Molecular dynamic (MD) studies of the highest affinity binder, **GMA 15**, were carried out to check its time-dependent stability with the receptor compared to DPA-714 and PK11195. The hydrogen bond plot also indicated that **GMA 15** formed higher hydrogen bonds compared to DPA-714 and PK11195. We anticipate that further optimization to enhance the potency in a cellular assay needs to be followed, but our strategy of identifying potential TSPO binding novel scaffolds may open up a new avenue to develop novel TSPO ligands suited for potential molecular imaging and a wide range of therapeutic applications.

## 1. Introduction

As the aging population increases worldwide, diseases associated with central nervous system (CNS) damage constitute an increasing public health concern. Alzheimer’s disease (AD), Parkinson’s disease (PD), multiple sclerosis (MS) and diseases related to ischemic brain injury or neurodevelopmental disorders are examples of CNS pathologies that do not have efficient treatments to prevent their progression. Neuroinflammation has been identified as a potential indicator for the diagnosis and treatment of neurodegenerative diseases, specifically, AD, PD and MS [1]. The translocator protein 18kDa (TSPO) is a highly lipophilic, tryptophan-rich protein (169 amino acids) with a five-membrane α-helices domain usually located in the outer mitochondrial membrane [2], where they combine with a 32 kDa voltage-dependent anion channel (VDAC) and a 30 kDa adenine nucleotide carrier (ANC) [3]. This trimeric complex, which is involved in the mitochondrial permeability transition pore (MPTP), plays an important role in certain transport processes. The TSPO controls the translocation of cholesterol from the outer to the inner mitochondrial membrane and subsequent enzymatic side chain cleavage to form pregnenolone, a key intermediate for the biosynthesis of steroids [4]. The TSPO is minimally produced in healthy brains, while it is overexpressed in inflamed brains. The overexpression of the TSPO is associated with microglial activation caused by neuronal damage or neuroinflammation, and these activated microglia are involved in various CNS diseases that are known or suspected to involve inflammation, such as AD, PD, MS and strokes [5]. The TSPO is also overexpressed in certain cancers, such as glioma and breast cancer, in which the expressed protein is linked with disease progression and is associated with aggressive phenotypes [6]. In the case of neuroblastoma, TSPO ligands induce apoptosis and cell cycle arrest, and offer sensitization to chemotherapy [7]. Several synthetic TSPO ligands were primarily developed as neuroimaging agents and diagnostic tools for the treatment of brain inflammation associated with neurodegenerative diseases [8].

As such, today the TSPO is a validated target for a number of therapeutic applications for neurological and psychiatric disorders [8,9], neurodegenerative diseases, traumatic brain injuries and strokes [10]; a therapeutic target in neurotrauma [11]; and a highly investigated target for the development of neuroprotective drugs [8,10]. Moreover, the TSPO is a well-recognized imaging biomarker for the evaluation of inflammation-related diseases and allows the level of brain inflammation to be quantified in vivo in a non-invasive way via positron emission tomography (PET) [12].

**Figure 1:**
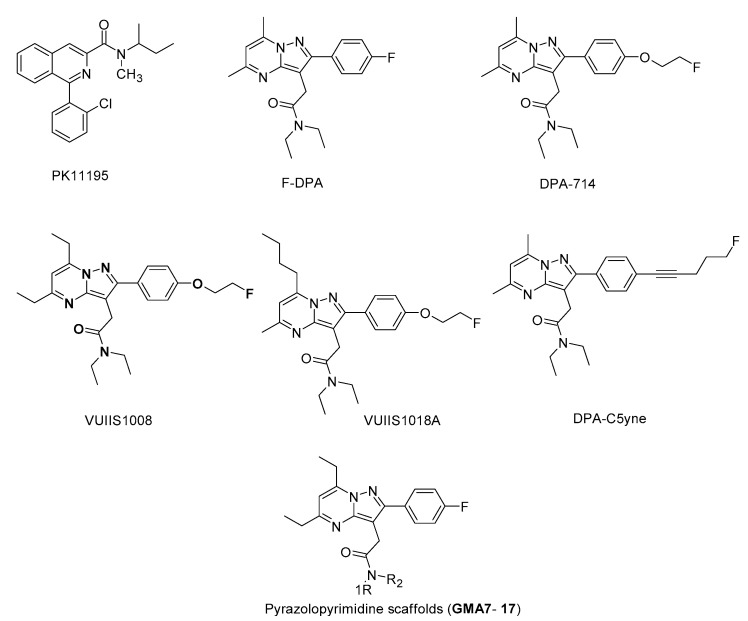
Selected high-affinity TSPO ligands and the synthesized pyrazolopyrimidine scaffold (**GMA 7**–**17**).

Several classes of TSPO ligands with high binding affinity and specificity have been reported to date (Figure 1) [13,14,15,16,17,18]. The first generation of radioligands, Ro5-4864 and PK11195, have shown their efficacy in reducing the level of microglia activation and the production of pro-inflammatory cytokines [19]. [^11^C]PK11195 has long been used in clinical studies to detect the human TSPO in vivo with PET [20]. However, its non-specific binding and slow pharmacokinetic properties are limitations for in vivo quantification of inflammatory processes in the brain. In view of these deficiencies, many studies have been conducted to develop novel TSPO radiotracers, including [^11^C]PBR28, [^11^C]DAA1106, [^18^F]PBR06, imidazolopyrimidine derivative [^11^C]CLINME and pyrazolopyrimidine derivative ([^18^F]DPA-714) ligands, which are selective for the TSPO and show more favorable characteristics in vivo [18]. Among these, the second-generation PET radioligand, [^18^F]DPA-714, is a pyrazolopyrimidinal precursor featuring a high binding affinity to TSPO [21]. Pyrazolopyrmidine acetamide (DPA-714) represents a particularly attractive target class of TSPO-specific ligands with high in vitro potency and human in vivo imaging studies [22]. Comparative evaluation of the TSPO radioligands [^18^F]DPA-714 and (*R*)-[^11^C]PK11195 in a neuroinflammation rat model suggested that [^18^F]DPA-714 is better for PET imaging than(*R*)-[^11^C]PK11195, as it showed better bioavailability to brain tissue, together with a low level of nonspecific binding and increased binding potential [23]. Furthermore, the number of nitrogen atoms in the central hetero atomic core of DPA-714 can impact the TSPO binding affinity [24]. Based on the scaffold of DPA-714, a variety of novel radioligands have been developed and used in TSPO PET imaging, including [^18^F]VUIIS1008 [24,25], [^18^F]DPA-C5yne [26] and [^18^F]VUIIS108A [16]. All these radioligands showed promising results in the in vivo dynamic PET imaging (Figure 1).

We have focused on the development and application of high-affinity TSPO ligands applicable to neuroinflammatory imaging modalities. We recently reported the phenoxypyrimidal acetamide [27] and pyrazolopyrimidine acetamide derivatives targeting the TSPO [28].

In this context, we aimed to design pyrazolopyrimidine acetamide derivatives (**GMA** scaffolds) and investigate their affinity and selectivity for the TSPO, both for therapeutic and diagnostic purposes. We are already aware of modified scaffolds that can obtain improved binding affinity through studies of FDPA and VUIIS1008, and we expected that these modifications would show higher binding affinity than DPA-714. In addition, we attempted to investigate whether the binding affinity would be improved when substituents of the amide structure, which had not received much attention before, had various structures and asymmetry. The novel *N*,*N*-disubstituted pyrazolopyrimidine acetamides scaffold (**GMA 7**–**17**), which bears a fluorine atom and is directly linked to the phenyl moiety, was synthesized. All of the new compounds were designed with a fluorine atom in their structure at a position suitable for further application to fluorine-18 labeling. Herein, we report the synthesis and initial in vitro biological evaluation of these new pyrazolopyrimidine scaffolds (**GMA 7**–**11**), with the aim to explore the pharmacophoric model within pyrazolopyrimidine acetamides for optimal binding to the TSPO.

## 2. Results and Discussion

### 2.1. Chemistry

**Scheme 1:**
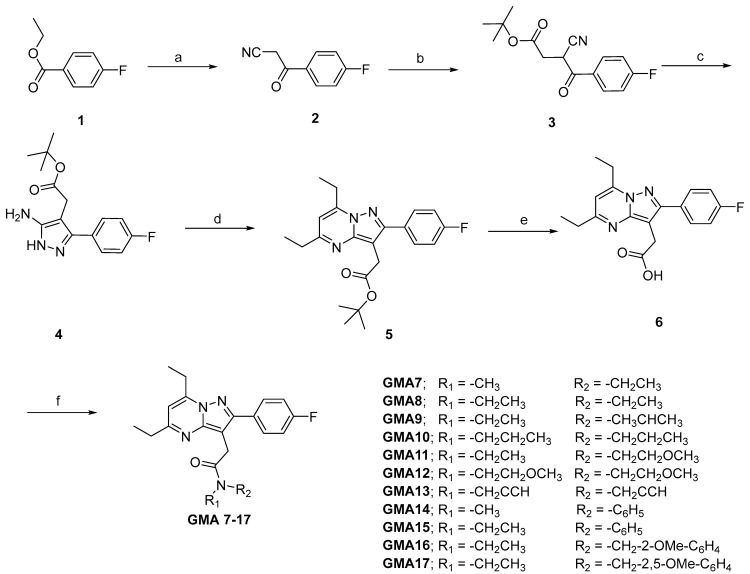
Reagents and conditions: (**a**) n-BuLi, ACN, −60 °C, 2 h, 45%; (**b**) tert-butyl 2-chloroacetate, K_2_CO_3_, NaI, THF, 5 h, r.t, 68%; (**c**) hydrazine, AcOH, EtOH, reflux, 8 h, 62%; (**d**) heptane-3,5-dione, EtOH, sealed tube, 90 °C, 6 h, 72%; (**e**) KOH, EtOH, reflux, 6 h, 76%; (**f**) HNR_1_R_2_, HATU, DIEPA, DMF, r.t, 10 h, 70–82%.

The target compounds in this study were synthesized as illustrated in Scheme 1. The synthetic route commenced with the commercially available compound methyl 4-fluorobenzoate (**1**) with a nucleophilic addition of acetonitrile to generate 3-(4-fluorophenyl)-3-oxopropanenitrile (**2**) upon the replacement of the ethoxide moiety. Initially, the reaction was performed with NaOMe in boiling acetonitrile with a poor yield of 26%. The reaction yield was improved by using n-BuLi at −60 °C to afford a modest yield of 45% [29]. *β*-ketonitrile (**2**) was C-alkylated with tert-butyl 2-chloroacetate in the presence of NaI in ethanolic sodium hydroxide to afford crude (**3**) [17], which was subjected to a first cyclization using monohydrated hydrazine in the presence of acetic acid to afford aminopyrazole (**4**) [14]. Compound **4** was reacted with 3,5-heptanedione by heating them in a sealed tube in ethanol to generate a close pyrimidine ring (**5**), followed by deprotection with NaOH to reveal the free carboxylic acid (**6**). Compound **6** was a key intermediate, as it could give access to a wide range of acetamide compounds in a straightforward manner. Finally, a variety of secondary amines were subsequently reacted with carboxylic acid **6** to generate the final products (**GMA 7**–**17**) evaluated here. In total, 11 analogs were synthesized with a diversity of *N*-acetamides substituents.

**Figure 2:**
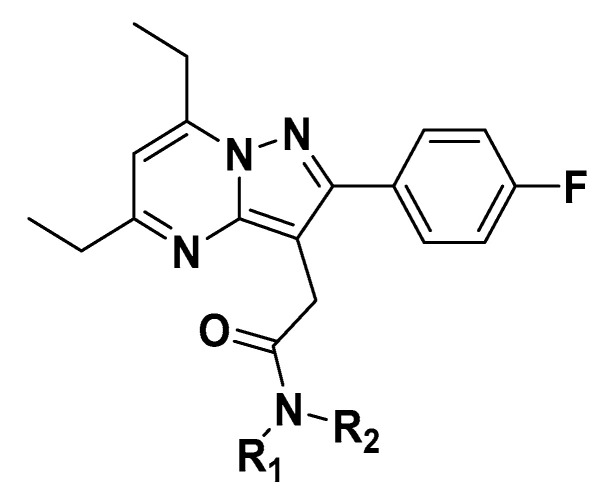
Chemical structure of novel pyrazolopyrimidine derivatives.

Upon synthesis and analytical characterization, the TSPO binding affinities (*Ki*) of the newly synthesized pyrazolopyrimidine derivatives, (**GMA 7**–**17**, Figure 2), PK11195 and DPA-714, were measured by competition with [^3^H]PK11195 in a membrane of human leukocytes. The selectivity of several pyrazolopyrimidine analogs for the TSPO were shown to surpass the selectivity of the central benzodiazepine receptor (CBR), as well as numerous other common targets [8,30]. The off-target activity levels of these novel pyrazolopyrimidine analogs were not determined, as the aim of the present study was to explore pyrazolopyrimidine SARs specifically for the replacement of related *N*,*N*-disubstitutions of the terminal acetamide, and the fluorine atom is directly linked to the phenyl moiety without the presence of an alkyl or alkoxy spacer chain. The affinities of compounds expressed as *Ki* (nM) values are presented in Table 1, and all the synthesized compounds displayed a picomolar to nanomolar binding affinity for the TSPO, with *Ki* values ranging from 0.06 to 25.37 nM. Within this series, *N*,*N*-di (2-methoxy ethyl) acetamide (**GMA 12**), *N*-ethyl, *N*-isopropyl acetamide (**GMA 9**) and *N*-Methyl-*N*-ethyl acetamide (**GMA 7**) showed similar values (25.37, 13.27 and 17.10 nM, respectively), which were similar to the values of DPA-714 (3.66 nM) and PK11195 (1.34 nM). It can be seen that shortening of the *N*,*N*-dialkyl substituents in the groups in the nitrogen amide is not beneficial to TSPO binding. TSPO binding was improved by the introduction of *N*,*N*-dipropargyl acetamide (**GMA 13**), which displayed nanomolar affinity (*Ki* = 0.90 nM). The *N*,*N*-dipropyl acetamide (**GMA 10**), *N*-ethyl and *N*-methoxy ethyl acetamide (**GMA 11**) compounds showed potential binding affinity (*Ki* = 0.18 and 0.19 nM). However, increasing the length of the *N*-alkyl chain had little effect on the TSPO binding affinity, which suggests that there may be a lipophilic binding pocket that can accommodate the steric *N*-alkyl ether. Interestingly, the introduction of the *N*-substituted aromatic substituent with the ethyl group was shown to be particularly favorable, resulting in the novel ligand **GMA 15** with picomolar activity of *Ki* = 60 pM, which is a dozens of times higher affinity compared to DPA-714 and PK11195 (61- and 22-fold, respectively). It was found that **GMA 15** had the highest binding in affinity compared to all the other compounds investigated. The presence of an aromatic moiety may have been advantageous due to a potential hydrophobic π–π interaction with a tryptophan residue in the binding pocket. Furthermore, the replacement of the *N*-aromatic with the *N*-benzyl substituent led to a decrease in affinity (0.49 to 0.54 nM), probably due to an unfavorable steric interaction with a lipophilic binding pocket of protein.

### 2.2. Molecular Docking, Molecular Dynamic and In Silico ADMET Studies

**Figure 3:**
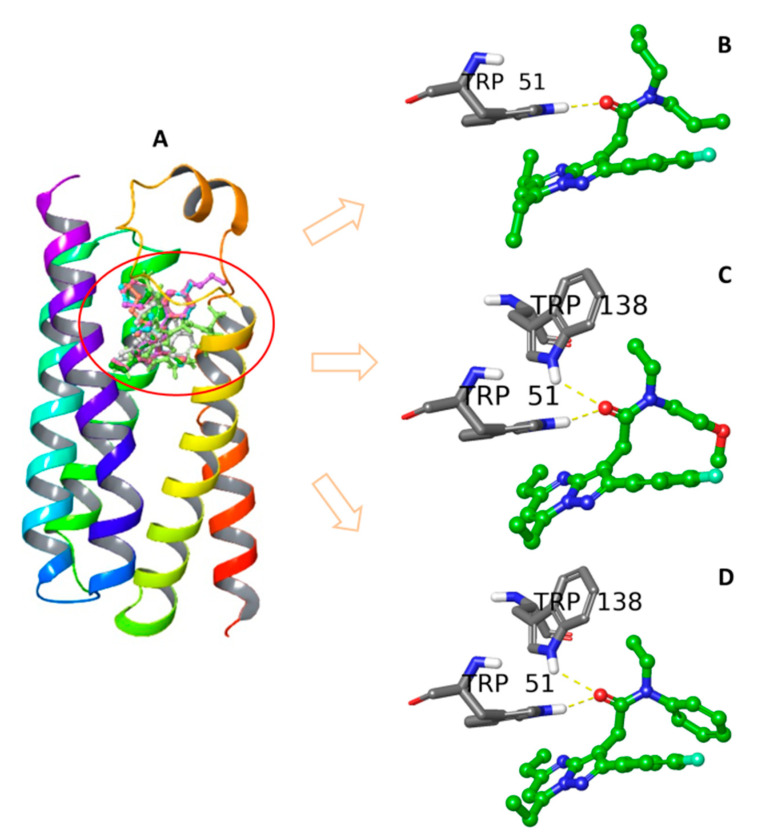
TSPO residues that form the ligand-binding pocket. The most favorable position for ligand binding is shown in green space-filling surface representation (**A**); **GMA 10** was docked onto Translocator proteins (BcTSPO) receptor with a docking score of −9.206, and an H-bond of length 2.6 Å to the binding pocket residue Trp-51 was observed (**B**); **GMA 11** was docked onto Translocator proteins (BcTSPO) receptor with a docking score of −8.988, and two H-bonds of length 2.5 and 2.2 Å to the binding pocket residue Trp-138 and Trp-51 were observed (**C**); **GMA 15** was docked onto Translocator proteins (BcTSPO) receptor with a docking score of −12.443, and two H-bonds of length 2.7 and 2.1 Å to the binding pocket residue Trp-138 and Trp-51 were observed (**D**).

We reported the three compounds with the highest docking score and analyzed their mode of interaction with the TSPO. These compounds exhibited better binding affinity than the validated *Bacillus cereus* TSPO (BcTSPO)/PK11195 ligand complex (Figure 3A) [31], which suggests the improved ligand binding power of these new compounds (Table 2). The docking results for **GMA 10** against BsTSPO (PDB ID: 4RYI) showed a high binding affinity docking score of −9.206, and two H-bonds, 2.6 Å in length, formed with hydrophobic residue, that is, Tryptophan-51. In the docking pose, the chemical natures of binding site residues within a radius of 3 Å from the bound compound were basic (polar, hydrophobic and positively charged), that is, Ile-47, Ile-145, Ile-27, Ile-149 (isoleucine), Ala-142 (alanine), Pro-42 (proline) and Val-110 (valine); aromatic (hydrophobic), that is, Phe-55, Phe-141, Phe-90 (phenylalanine), Trp-51 (tryptophan) and Tyr-32 (tyrosine); nucleophilic (polar and hydrophobic), that is, Ser-146, Ser-91, Ser-21, Ser-22 (serine) and Cys-107(cysteine); and polar amide, that is, Asn-87 (asparagine); thus, the bound compound showed high binding affinity and strong hydrophobic interaction, which may have led to improved binding stability and affinity (Figure 3B). Similarly, the docking results for **GMA 11** against the TSPO showed a high binding affinity docking score, which was −8.988, and H-bonds with lengths of 2.5 and 2.2 Å formed with hydrophobic residue, that is, Tryptophan-51 and Tryptophan-138. In the docking pose, the chemical natures of binding site residues within a radius of 3 Å from the bound compound were aromatic (hydrophobic), that is, Phe-55, Phe-141, Phe-90, Phe-95 (phenylalanine), Trp-51, Trp-138 (tryptophan), Tyr-32 and Tyr-135 (tyrosine); polar amide, that is, Asn-87 (asparagine) and Gln-94 (glutamine); basic (polar, hydrophobic and positively charged), that is, Ile-27, Ile-47, Ile-145, Ile-149 (isoleucine), Ala-142 (alanine), Pro-42 (proline) and Val-110 (valine); and nucleophilic (polar, hydrophobic), that is, Ser-91, Ser-21, Ser-146, Ser-22 (serine) and Cys-107(cysteine); thus, the bound compound showed high binding affinity and strong hydrophobic interaction, which may have led to improved binding stability and affinity (Figure 3C). Likewise, the docking results for **GMA 15** against the TSPO showed that **GMA 15** had the highest affinity, with an indicated ligand docking score of −12.443, and H-bonds with lengths of 2.5 and 2.1 Å formed with hydrophobic residue, that is, Tryptophan-51 and Tryptophan-138. In the docking pose, the chemical natures of the binding site residues within a radius of 3 Å from the bound compound were polar amide, that is, Asn-87 (asparagine); nucleophilic (polar and hydrophobic), that is, Ser-22, Ser-21, Ser-91, Ser-139, Ser-146 (serine) and Cys-107 (cysteine); aromatic (hydrophobic), that is, Phe-55, Phe-141, Phe-90 (phenylalanine), Trp-135, Trp-51, Trp-138 (tryptophan), Tyr-32 and Tyr-135 (tyrosine); and basic (polar, hydrophobic and positively charged), that is, Val-110 (valine), Leu-145 (leucine), Ile-27, Ile-47, Ile-145, Ile-149 (isoleucine), Ala-142 (alanine) and Pro-42 (proline); thus, the bound compound showed high binding affinity and strong hydrophobic interaction, which may have led to higher stability and binding affinity (Figure 3D).

The docking results for the control compounds, DPA-714 and PK11195 (TSPO inhibitors), with the translocator proteins showed low and similar binding affinity scores, indicated by −8.252 and −10.083, and they showed that an H-bond formed with hydrophobic residue, that is, Tryptophan-51 and Tryptophan-138. In comparison, the docking scores of the new synthesized pyrazolopyrimidine scaffolds, **GMA 10**, **GMA 11** and **GMA 15**, showed total scores of −9.206, −8.988 and −12.443, respectively (Table 2). Thus, the docking procedure in Glide software used to reproduce the experimental binding affinity seems reliable and was therefore predicted as a true positive.

**Figure 4:**
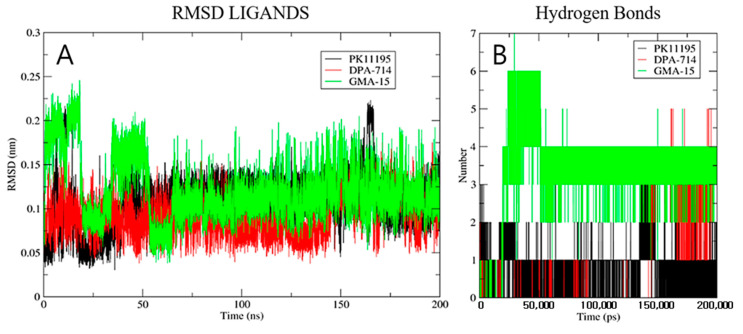
MD simulation results: (**A**) Indicates RMSD plots of the **GMA 15** ligand compared to PK11195 and DPA-714. (**B**) Indicates hydrogen bond interactions of **GMA 15** compared to PK11195 and DPA-714.

Finally, based on the computed docking Glide score, those with the best poses were chosen and ranked based on their E model value [32]. Various reports have indicated the use of molecular dynamics for the prediction of the stability of protein ligand complexes [33,34,35]. We carried out MD studies on the top molecule, **GMA 15**, to determine its time-dependent stability with the receptor compared to DPA-714 and PK11195. In the 200 ns MD run, compared to PK11195, **GMA 15** had minor stable deviations of less than 2 Å (Figure 4A). We observed that the compound DPA-714 was also stable in the RMSD plot, but formed fewer hydrogen bonds. The hydrogen bond plot also indicated that **GMA 15** formed six hydrogen bonds compared to DPA-714, and PK11195, which only formed five and three hydrogen bonds (Figure 4B). This indicated that the compound **GMA 15** might have a better interaction profile and better potency then both PK11195 and the core scaffold DPA-714. The visualized docking information in detail is provided at supplementary material.

The lead compound, **GMA 15**, was further subjected to in silico ADMET (absorption, distribution, metabolism, excretion and toxicity) studies, which found **GMA 15** to have good blood–brain barrier (BBB) penetration; have good Human Intestinal Absorption (HIA); be non-toxic and non-carcinogenic in the AMES test; be a weak inhibitor of a human ether-a-go-go-related gene (hERG); and be capable of being absorbed through the gastrointestinal tract (Table S1) [36,37]. **GMA 15** showed good HIA and BBB properties, indicating its better permeability and absorption behavior. Furthermore, the AMES non-toxicity and non-carcinogenicity test helped to identify the potential mutagenic and cancer-causing properties in **GMA 15**. The information of physicochemical data, ADMET predictions, cytotoxicities for five compounds in detail are provided at supplementary material.

## 3. Materials and Methods

### 3.1. Chemistry

All solvents were purified and used in scrupulously dry conditions. The NMR spectra of all compounds were recorded with a Bruker AC-400 spectrometer (400 MHz for ^1^H and 100 MHz for ^13^C). Chemical shifts were reported as values in parts per million (ppm) and coupling constants (J) in Hz. Multiplicities were described as s (singlet), d (doublet), t (triplet), q (quartet), m (multiplet) and dd (doublet of doublets), downfield from the internal TMS standard. All the air- and moisture-sensitive reactions were kept under inert conditions, and the reactions were monitored via thin-layer chromatography (TLC) using Merck silica gel 60F–254 plates, and chromatography was performed using Merck Kiesegel 60 Art 9385 (230–400 mesh). Mass spectra (MS) were recorded with Advion expression mass spectroscopy.

#### General Experimental Information

3-(4-Fluorophenyl)-3-oxopropanenitrile (**2**): *n*-BuLi (1.3 eq) was added to a solution of acetonitrile (1.3 eq) and THF at −60 to 65 °C, then the reaction mixture was stirred for 30–45 min, and Methyl 4-fluorobenzoate (**1**, 1.0 eq) in THF was slowly added to the above mass at −60 to 65 °C. The reaction was maintained at −60 to 65 °C for 2 h. Saturated sodium chloride solution was added to the reaction mixture and extracted with ethyl acetate (100 mL × 3 times), separated into organic layers and dried over Na_2_SO_4_, and concentrated to get the crude mass, which was purified with column chromatography using a 1:4 to 1:2 (*v/v*) ethyl acetate and petroleum ether solution as an eluent to afford the *β*-ketonitriles light-yellow solid compound in a 45% yield. ^1^H-NMR (MeOD, 400 MHz) δ 7.98–7.91 (m, 1H), 7.23–7.17 (m, 2H), 4.08 (s, 2H).

*Tert*-butyl 3-cyano-4-(4-fluorophenyl)-4-oxobutanoate (**3**): To a solution of 3-(4-methoxy phenyl)-3-oxopropanenitrile (**2**, 1 eq) and tert-butyl 2-bromoacetate (1.2 eq) in THF (10 mL) was added K_2_CO_3_ (1.3 eq) and NaI (0.5 eq). The reaction mixture was stirred at 25–30 °C for 5 h. The reaction mass was poured into water and extracted with ethyl acetate (100 mL × 3 times), separated into organic layers and dried over Na_2_SO_4_, and concentrated to get the crude mass and afford the compound in a 68% yield. ^1^H-NMR (MeOD, 400 MHz) δ 7.98 (d, *J* = 8.8 Hz, 1H), 7.84 (d, *J* = 11.6 Hz, 2H), 7.32–7.28 (m, 1H), 3.96 (d, *J* = 10.6 Hz, 1H), 3.09 (d, *J* = 16.8 Hz, 1H), 2.92 (d, *J* = 16.8 Hz, 1H), 1.48 (s, 9H).

*Tert*-butyl 2-(5-amino-3-(4-fluorophenyl)-1H-pyrazol-4-yl)acetate (**4**): To a solution of *tert*-butyl 3-cyano-4-(4-flurophenyl)-4-oxobutanoate (**3**, 1 eq) in ethanol was added hydrazine hydrate (2 eq) and acetic acid (1 eq). The reaction mixture was refluxed for 8–10 h. After cooling, the solvent was evaporated under reduced pressure to obtain a residue, and the residue was dissolved in dichloromethane, washed with water, and the organic layer was dried over Na_2_SO_4_ and concentrated to get a syrup mass. Yield 62%; ^1^H-NMR (MeOD, 400 MHz) δ 7.35–7.31 (m, 2H), 7.20–7.15 (m, 2H), 3.95–3.92 (m, 2H), 1.34 (s, 9H).

*Tert*-butyl 2-(5,7-diethyl-2-(4-fluorophenyl)pyrazolo [1,5-a]pyrimidin-3-yl)acetate (**5**): To a solution of *tert*-butyl 2-(5-amino-3-(4-flurophenyl)-1H-pyrazol-4-yl) acetate (**4**, 1 eq) in ethanol was added heptane-3,5-dione (1.1 eq) in a sealed tube. The reaction mixture was heated to 80–90 °C for 8–10 h. After cooling, the solvent was evaporated under reduced pressure to obtain a residue, which was purified with column chromatography using a 2:4 to 3:4 (*v/v*) ethyl acetate and petroleum ether solution as an eluent to afford **5**. Yield 72%; 1H-NMR (MeOD, 400 MHz) δ 7.43 (t, *J* = 8.8 Hz, 2H,), 7.10 (t, *J* = 8.8 Hz, 2H), 6.70 (s, 1H), 3.82 (s, 2H), 3.10 (dd, *J* = 14.6 Hz, 7.6 Hz, 2H), 2.76 (dd, *J* = 14.6 Hz,7.6 Hz, 2H), 1.36 (t, *J* = 7.6 Hz, 3H), 1.34 (s, 9H), 1.26 (t, *J* = 7.6 Hz, 3H).

2-(5,7-Diethyl-2-(4-fluorophenyl)pyrazolo [1,5-a]pyrimidin-3-yl)acetic acid (**6**): To a solution of *tert*-butyl 2-(5,7-diethyl-2-(4-flurophenyl)pyrazolo [1,5-a]pyrimidin-3-yl)acetate (**5**, 1 eq) in ethanol (10 mL ) was added KOH (2 eq) and water (3 mL), and they were added to a sealed tube. The reaction mixture was heated to 80–90 °C for 8 h. After cooling, the solvent was evaporated under reduced pressure, and the residue was dissolved in water acidified with 1.0 M aqueous HCl and extracted with dichloromethane three times. The organic solutions were then collected, dried over sodium sulfate, and concentrated to dryness to get a solid mass. Yield 76%; ^1^H-NMR (MeOD, 400 MHz) δ 7.45 (t, 2H, *J* = 8.8 Hz), 7.11 (t, 2H, *J* = 8.8 Hz), 6.72 (s, 1H), 3.83 (s, 2H), 3.11 (dd, 2H, *J* = 14.6 Hz, *J* = 7.6 Hz), 2.78 (dd, 2H, *J* = 14.6 Hz, *J* = 7.6 Hz), 1.37 (t, 3H, *J* = 7.6 Hz), 1.26 (t, 3H, *J* = 7.6 Hz).

General Procedure A:

To a solution of 2-(5,7-Diethyl-2-(4-fluorophenyl)pyrazolo [1,5-a]pyrimidin-3-yl)acetic acid (1 eq) in DMF (5 mL) was added HATU (1.2 eq), *N*,*N*-diisopropylethylamine (2.5 eq) and 1.1 eq of *N*-alkyl/aryl amine. The reaction mixture was then maintained for 10–12 h at r.t. The reaction was followed by TLC to completion, after which the mixture was then purified with column chromatography using a 2:4 to 3:1 (*v/v*) ethyl acetate and petroleum ether solution as an eluent to afford the compound as a syrup mass, and the mass was triturated with petroleum ether to get a solid mass in 70–85% yields.

2-(5,7-diethyl-2-(4-fluorophenyl)pyrazolo [1,5-a]pyrimidin-3-yl)-*N*-ethyl-*N*-methylacetamide (**GMA 7**): **GMA 7** (40 mg) was obtained according to the general procedure A from **6** (50 mg, 0.15 mmol), HATU (69 mg, 0.18 mmol), DIEPA (48 mg, 0.37 mmol) and *N*-ethyl-2-methoxyethanamine (21 mg, 0.21 mmol). Yield 72%; ^1^H NMR (600 MHz, DMSO) δ 7.84 (m, 2H), 7.37–7.23 (m, 2H), 6.87 (s, 1H), 3.86 (d, *J* = 11.8 Hz, 2H), 3.56 (q, *J* = 7.1 Hz, 1H), 3.37–3.25 (m, 2H), 3.19–3.05 (m, 3H), 2.85–2.74 (m, 3H), 1.37 (t, *J* = 7.5 Hz, 3H), 1.26 (t, *J* = 7.6 Hz, 3H), 1.18 (t, *J* = 7.1 Hz, 2H), 0.97 (t, *J* = 7.1 Hz, 2H). ^13^C NMR (151 MHz, DMSO) δ 170.00, 162.93, 161.92, 153.25, 149.94, 147.59, 130.62, 115.96, 106.33, 101.56, 44.39, 42.48, 35.19, 33.14, 31.11, 28.05, 27.45, 23.29, 13.85, 12.96, 10.72. MS (ESI) *m/z* (M + Na)+ 391.23.

2-(5,7-diethyl-2-(4-fluorophenyl)pyrazolo [1,5-a]pyrimidin-3-yl)-*N*,*N*-diethylacetamide (**GMA 8**): **GMA 8** (45 mg) was obtained according to the general procedure A from **6** (50 mg, 0.15 mmol), HATU (69 mg, 0.18 mmol), DIEPA (48 mg, 0.37 mmol) and diethylamine (21 mg, 0.21 mmol). Yield 82%; ^1^H NMR (600 MHz, DMSO) δ 7.91–7.78 (m, 2H), 7.36–7.23 (m, 2H), 6.87 (t, *J* = 0.9 Hz, 1H), 3.85 (s, 2H), 3.54 (q, *J* = 7.1 Hz, 2H), 3.26 (q, *J* = 7.0 Hz, 2H), 3.16–3.05 (m, 2H), 2.79 (q, *J* = 7.5 Hz, 2H), 1.37 (t, *J* = 7.4 Hz, 3H), 1.26 (t, *J* = 7.5 Hz, 3H), 1.18 (t, *J* = 7.1 Hz, 3H), 0.98 (t, *J* = 7.0 Hz, 3H). ^13^C NMR (151 MHz, DMSO) δ 169.56, 162.86, 161.91, 153.24, 149.89, 147.56, 130.60, 130.55, 130.52, 115.94, 115.79, 106.33, 101.66, 42.21, 40.53, 31.04, 27.68, 23.29, 14.78, 13.55, 12.97, 10.73. MS (ESI) *m/z* (M + Na)+ 405.21.

2-(5,7-Diethyl-2-(4-fluorophenyl)pyrazolo [1,5-a]pyrimidin-3-yl)-*N*-ethyl-*N*-isopropyl acetamide (**GMA 9**): **GMA 9** (46 mg) was obtained according to the general procedure A from **6** (50 mg, 0.15 mmol), HATU (69 mg, 0.18 mmol), DIEPA (48 mg, 0.37 mmol) and *N*-ethylpropan-2-amine (20 mg, 0.21 mmol). Yield 75%; ^1^H NMR (600 MHz, DMSO) δ 7.85 (m, 2H), 7.30 (m, 2H), 6.87 (d, *J* = 5.7 Hz, 1H), 4.52–4.35 (m, 1H), 3.88 (s, 1H), 3.83 (s, 1H), 3.49 (q, *J* = 7.1 Hz, 1H), 3.21–2.99 (m, 3H), 2.79 (q, *J* = 7.5 Hz, 2H), 1.37 (td, *J* = 7.5, 1.0 Hz, 3H), 1.32–1.19 (m, 5H), 1.15 (d, *J* = 6.6 Hz, 3H), 1.07 (d, *J* = 6.8 Hz, 3H), 0.96 (t, *J* = 6.9 Hz, 2H). ^13^C NMR (151 MHz, DMSO) δ 170.03, 162.92, 153.25, 149.95, 147.58, 130.63, 115.93, 106.34, 101.98, 48.20, 45.61, 37.64, 35.37, 31.11, 28.23, 23.30, 21.51, 20.65, 16.97, 15.24, 13.17, 12.90, 10.73. MS (ESI) *m/z* (M + H)+ 433.36.

2-(5,7-diethyl-2-(4-fluorophenyl)pyrazolo [1,5-a]pyrimidin-3-yl)-*N*,*N*-dipropylacetamide (**GMA 10**): **GMA 10** (45 mg) was obtained according to the general procedure A from **6** (50 mg, 0.15 mmol), HATU (69 mg, 0.18 mmol), DIEPA (48 mg, 0.37 mmol) and dipropylamine (21 mg, 0.21 mmol). Yield 73%; ^1^H NMR (600 MHz, DMSO) δ 7.93–7.70 (m, 2H), 7.39–7.19 (m, 2H), 6.86 (d, *J* = 0.9 Hz, 1H), 3.87 (s, 2H), 3.55–3.37 (m, 2H), 3.29 (d, *J* = 14.9 Hz, 5H), 3.22–3.16 (m, 2H), 3.12 (qd, *J* = 7.5, 0.8 Hz, 2H), 2.79 (q, *J* = 7.5 Hz, 2H), 1.60 (h, *J* = 7.4 Hz, 2H), 1.39 (dt, *J* = 20.2, 7.5 Hz, 5H), 1.26 (t, *J* = 7.6 Hz, 3H), 0.86 (t, *J* = 7.4 Hz, 3H), 0.75 (t, *J* = 7.4 Hz, 3H). ^13^C NMR (151 MHz, DMSO) δ 170.09, 162.83, 153.23, 149.89, 147.52, 130.63, 130.52, 130.50, 115.90, 115.76, 106.35, 101.70, 49.55, 47.62, 40.52, 31.03, 27.91, 23.28, 22.38, 20.99, 12.90, 11.63, 11.47, 10.73. MS (ESI) *m/z* (M + Na)+ 433.22.

2-(5,7-diethyl-2-(4-fluorophenyl)pyrazolo [1,5-a]pyrimidin-3-yl)-*N*-ethyl-*N*-(2-methoxyethyl) acetamide (**GMA 11**): **GMA 11** (48 mg) was obtained according to the general procedure A from **6** (50 mg, 0.15 mmol), HATU (69 mg, 0.18 mmol), DIEPA (48 mg, 0.37 mmol) and *N*-ethyl-2-methoxyethanamine (23 mg, 0.21 mmol). Yield 77%; ^1^H NMR (600 MHz, DMSO) δ 7.97–7.68 (m, 2H), 7.35–7.16 (m, 2H), 6.87 (dd, *J* = 5.0, 1.1 Hz, 1H), 3.93 (s, 1H), 3.88 (s, 1H), 3.69 (t, *J* = 5.5 Hz, 1H), 3.62–3.48 (m, 2H), 3.40 (dd, *J* = 6.8, 5.4 Hz, 1H), 3.35 (dd, *J* = 6.1, 4.8 Hz, 1H), 3.30 (d, *J* = 16.1 Hz, 3H), 3.21 (s, 1H), 3.12 (q, *J* = 7.5 Hz, 2H), 2.80 (m, 2H), 1.38 (t, *J* = 7.5 Hz, 3H), 1.27 (m, 3H), 1.18 (t, *J* = 7.1 Hz, 1H), 0.97 (t, *J* = 7.0 Hz, 1H). ^13^C NMR (151 MHz, DMSO) δ 170.23, 162.90, 153.23, 149.91, 147.65, 130.60, 115.95, 106.35, 101.71, 71.08, 70.38, 58.92, 58.52, 47.28, 45.37, 43.50, 41.10, 31.08, 27.99, 23.29, 14.52, 13.24, 10.73. MS (ESI) *m/z* (M + H)+ 435.22.

2-(5,7-Diethyl-2-(4-fluorophenyl)pyrazolo [1,5-a]pyrimidin-3-yl)-*N*,*N*-bis(2-methoxyethyl) acetamide (**GMA 12**): **GMA 12** (52 mg) was obtained according to the general procedure A from **6** (50 mg, 0.15 mmol), HATU (69 mg, 0.18 mmol), DIEPA (48 mg, 0.37 mmol) and bis(2-methoxyethyl)amine (25 mg, 0.21 mmol). Yield 77%; ^1^H NMR (600 MHz, DMSO) δ 7.94–7.67 (m, 2H), 7.39–7.20 (m, 2H), 6.87 (s, 1H), 3.97 (s, 2H), 3.74 (t, *J* = 5.4 Hz, 2H), 3.54 (t, *J* = 5.4 Hz, 2H), 3.34 (m, 5H), 3.31 (s, 3H), 3.16–3.07 (m, 2H), 2.80 (m, 2H), 1.38 (t, *J* = 7.5 Hz, 3H), 1.27 (t, *J* = 7.6 Hz, 3H). ^13^C NMR (151 MHz, DMSO) δ 170.87, 162.87, 153.08, 149.90, 147.68, 130.57, 130.47, 130.42, 115.90, 115.76, 106.31, 101.58, 70.94, 70.30, 58.92, 58.54, 48.47, 45.92, 40.53, 31.08, 27.97, 23.30, 13.07, 10.74. MS (ESI) *m/z* (M + Na)+ 465.22

2-(5,7-diethyl-2-(4-fluorophenyl)pyrazolo [1,5-a]pyrimidin-3-yl)-*N*,*N*-di(prop-2-yn-1-yl) acetamide (**GMA 13**): **GMA 13** (46 mg) was obtained according to the general procedure A from **6** (50 mg, 0.15 mmol), HATU (69 mg, 0.18 mmol), DIEPA (48 mg, 0.37 mmol) and dipropargylamine (23 mg, 0.21 mmol). Yield 76%; ^1^H NMR (600 MHz, DMSO) δ 7.94–7.68 (m, 2H), 7.40–7.16 (m, 2H), 6.90 (d, *J* = 1.0 Hz, 1H), 4.52 (d, *J* = 2.5 Hz, 2H), 4.17 (d, *J* = 2.5 Hz, 2H), 4.05 (s, 2H), 3.41 (t, *J* = 2.4 Hz, 1H), 3.31 (s, 1H), 3.23 (t, *J* = 2.5 Hz, 1H), 3.14 (qd, *J* = 7.5, 0.9 Hz, 2H), 2.80 (q, *J* = 7.6 Hz, 2H), 1.38 (t, *J* = 7.4 Hz, 3H), 1.27 (t, *J* = 7.6 Hz, 3H). ^13^C NMR (151 MHz, DMSO) δ 170.87, 162.87, 153.08, 149.90, 147.68, 130.57, 130.47, 130.42, 115.90, 115.76, 106.31, 101.58, 70.94, 70.30, 58.92, 58.54, 48.47, 45.92, 40.53, 31.08, 27.97, 23.30, 13.07, 10.74. MS (ESI) *m/z* (M + Na)+ 425.12.

2-(5,7-diethyl-2-(4-fluorophenyl)pyrazolo [1,5-a]pyrimidin-3-yl)-*N*-methyl-*N*-phenyl acetamide (**GMA 14**): **GMA 14** (42 mg) was obtained according to the general procedure A from **6** (50 mg, 0.15 mmol), HATU (69 mg, 0.18 mmol), DIEPA (48 mg, 0.37 mmol) and *N*-methylaniline (41 mg, 0.21 mmol). Yield 70%; ^1^H NMR (600 MHz, DMSO) δ 7.76 (t, *J* = 6.9 Hz, 2H), 7.46 (d, *J* = 7.7 Hz, 3H), 7.35 (t, *J* = 8.5 Hz, 3H), 6.85 (s, 1H), 3.60 (s, 2H), 3.32 (s, 2H), 3.17 (s, 2H), 3.10 (q, *J* = 7.5 Hz, 2H), 2.90–2.70 (m, 2H), 1.36 (td, *J* = 7.5, 1.2 Hz, 2H), 1.28 (td, *J* = 7.6, 1.2 Hz, 2H). ^13^C NMR (151 MHz, DMSO) δ 170.14, 163.54, 162.94, 161.91, 152.96, 149.87, 147.58, 144.35, 130.45, 130.39, 130.16, 127.85, 116.10, 115.95, 106.36, 101.25, 40.80, 40.53, 39.69, 37.67, 31.08, 28.90, 23.27, 13.06, 10.73. MS (ESI) *m/z* (M + Na)+ 439.12.

2-(5,7-diethyl-2-(4-fluorophenyl)pyrazolo [1,5-a]pyrimidin-3-yl)-*N*-ethyl-*N*-phenylacetamide (**GMA 15**): **GMA 15** (52 mg) was obtained according to the general procedure A from **6** (50 mg, 0.15 mmol), HATU (69 mg, 0.18 mmol), DIEPA (48 mg, 0.37 mmol) and *N*-ethylaniline (41 mg, 0.21 mmol). Yield 82%; ^1^H NMR (600 MHz, CDCl3) δ 7.84–7.69 (m, 2H), 7.39 (m, 2H), 7.31 (d, *J* = 7.5 Hz, 3H), 7.19–7.12 (m, 2H), 6.52 (s, 1H), 3.75 (q, *J* = 7.1 Hz, 2H), 3.68 (s, 2H), 3.17 (q, *J* = 7.5 Hz, 2H), 2.85 (t, *J* = 7.6 Hz, 2H), 1.44 (t, *J* = 7.5 Hz, 3H), 1.37 (t, *J* = 7.6 Hz, 3H), 1.11 (t, *J* = 7.1 Hz, 3H). ^13^C NMR (151 MHz, CDCl3) δ 170.21, 163,23, 162.43, 162.21, 162.06, 142.59, 142.23, 130.48, 130.43, 129.54, 128.55, 127.67, 115.50, 115.36, 115.09, 105,65, 105.09, 101.30, 44.49, 31.85, 29.42, 23.34, 13.06, 12.95, 12.65, 10.33. MS (ESI) *m/z* (M + Na)+ 453.22.

2-(5,7-diethyl-2-(4-fluorophenyl)pyrazolo [1,5-a]pyrimidin-3-yl)-*N*-ethyl-*N*-(2-methoxy benzyl)acetamide (**GMA 16**): **GMA 16** (51 mg) was obtained according to the general procedure A from **6** (50 mg, 0.15 mmol), HATU (69 mg, 0.18 mmol), DIEPA (48 mg, 0.37 mmol) and *N*-(2-methoxybenzyl)ethanamine (37 mg, 0.21 mmol). Yield 71%; ^1^H NMR (600 MHz, CDCl3) δ 7.89 (m, 2H), 7.29–7.03 (m, 5H), 6.88 (m, 2H), 6.52 (d, *J* = 32.9 Hz, 1H), 4.72 (d, *J* = 61.3 Hz, 2H), 3.99 (d, *J* = 31.5 Hz, 2H), 3.82 (d, *J* = 1.9 Hz, 3H), 3.61 (d, *J* = 7.1 Hz, 1H), 3.42 (q, *J* = 7.1 Hz, 1H), 3.26–3.08 (m, 2H), 2.85 (m, 2H), 1.44 (dt, *J* = 19.5, 7.5 Hz, 3H), 1.36 (dt, *J* = 12.9, 7.6 Hz, 3H), 1.23 (t, *J* = 7.1 Hz, 1H), 1.09 (t, *J* = 7.0 Hz, 2H). ^13^C NMR (151 MHz, CDCl3) δ 171.31, 162.52, 157.38, 156.91, 154.16, 130.73, 130.67, 128.76, 126.78, 125.96, 125.31, 120.57, 115.51, 110.06, 109.79, 105.19, 101.25, 55.30, 46.63, 43.11, 42.71, 31.38, 28.43, 27.66, 23.34, 14.10, 12.81, 10.33. MS (ESI) *m/z* (M + Na)+ 497.21.

2-(5,7-diethyl-2-(4-fluorophenyl)pyrazolo [1,5-a]pyrimidin-3-yl)-*N*-(2,5-dimethoxybenzyl)-*N*-ethylacetamide (**GMA 17**): **GMA 17** (54 mg) was obtained according to the general procedure A from **5** (50 mg, 0.15 mmol), HATU (69 mg, 0.18 mmol), DIEPA (48 mg, 0.37 mmol) and *N*-(2,5-dimethoxybenzyl)ethanamine (38 mg, 0.21 mmol). Yield 72%; ^1^H NMR (600 MHz, CDCl3) δ 8.03–7.77 (m, 2H), 7.21–7.04 (m, 2H), 6.85–6.69 (m, 2H), 6.64 (d, *J* = 2.7 Hz, 1H), 6.52 (dt, *J* = 32.2, 1.1 Hz, 1H), 4.70 (d, *J* = 59.9 Hz, 2H), 3.99 (d, *J* = 30.5 Hz, 2H), 3.77 (d, *J* = 4.1 Hz, 3H), 3.69 (d, *J* = 61.2 Hz, 3H), 3.61 (t, *J* = 7.1 Hz, 1H), 3.43 (q, *J* = 7.1 Hz, 1H), 3.26–3.09 (m, 2H), 2.84 (p, *J* = 7.8 Hz, 2H), 1.44 (dt, *J* = 18.9, 7.5 Hz, 3H), 1.34 (dt, *J* = 13.3, 7.6 Hz, 3H), 1.25 (t, *J* = 7.1 Hz, 2H), 1.11 (t, *J* = 7.1 Hz, 2H). ^13^C NMR (151 MHz, CDCl3) δ 171.28, 162.44, 153.78, 151.66, 130.65, 130.51, 129.96, 127.33, 126.71, 115.54, 114.99, 113.71, 112.28, 111.21, 110.49, 105.18, 101.12, 100.77, 55.98, 46.65, 42.97, 41.59, 31.37, 28.52, 27.70, 23.33, 14.07, 12.84, 10.32. MS (ESI) *m/z* (M + Na)+ 527.21.

### 3.2. Molecular Docking, Molecular Dynamic and In Silico ADMET Studies

#### 3.2.1. Molecular Docking

Molecular docking was performed using the docking tool GLIDE (Schrodinger Inc., LLC, New York, NY, USA, 2008) to study the ligand interaction in the BcTSPO receptor binding pocket. The crystal structure of the BcTSPO receptor complexed with the co-crystallized PK11195 inhibitor (PDB ID: 4RYI) with a resolution of 3.49 Å was obtained from the protein data bank. In this, PK11195 is bound by a tight bundle of five transmembrane helices that form a hydrophobic pocket. Hence, first we took the monomeric identified crystal structure of TSPO and explored the binding site of PK11195 to understand their prospective interactions with TSPO. The receptor molecule was prepared using two steps in the “protein preparation wizard” in the Maestro interface, and the molecule was refined for docking studies. The water molecules present in the receptor were removed, hydrogens were added, and energy minimization was carried out using the OPLS force field [38]. The Grid box was defined for the receptor by centering the existing co-crystallized ligand PK11195 [31]. The low-energy conformation of the ligands was prepared with the LigPrep module in Maestro using the OPLS force field. The ligands were docked in the receptor based on the grid using the extra precision (XP) docking algorithm to rank the ligand with a specific conformation of the receptor molecule [39].

#### 3.2.2. Molecular Dynamics Studies

Molecular dynamics studies were carried out using the Gromacs software [33]. We used the methodology employed in previous reports for setting up molecular dynamics studies for our molecules [34,35].

#### 3.2.3. In Silico Physicochemical and ADMET Studies

The physicochemical, absorption, distribution, metabolism, excretion and toxicity (ADMET) properties of the lead compound were predicted using the online tools SwisssADME (available at http://www.swissadme.ch, accessed on 15 January 2023) [36] and admetSAR1 (available at http://lmmd.ecust.edu.cn/admetsar1, accessed on 15 January 2023) [37].

#### 3.2.4. In Vitro Cytotoxicity Studies

The cytotoxicity analysis (MTT assay) for GMA 10, GMA 11, GMA 15, DPA-714 and PK11195 to L-929 (mouse fibroblast cells) was carried out. Five concentrations of each compound dissolved in 2% of DMSO in culture media (50 μL) were introduced into cell media (96-well plate). The cell cultures treated with ligands were incubated 24 h, and then the media were removed. The cell crudes were measured at 570 nm and 650 nm using a MTT reagent. All procedures and reagents followed ISO 10993-5 and ISO 10993-12.

### 3.3. In Vitro TSPO Binding Assay

The prepared TSPO binding compounds, **GMA 7**–**17**, were assayed for their binding affinity to TSPO using isolated human leukocytes and [^3^H]PK11195. These experiments were carried out with the previously reported method [40,41].

## 4. Conclusions

A new series of *N*,*N*-disubstituted pyrazolopyrimidine acetamide scaffolds (**GMA 7**–**17**), which bear fluorine atoms and are directly linked to the phenyl moiety with variation at *N*,*N*-substitutions of alkyl or aryl at the terminal acetamide, was synthesized, and the in vitro characterization of each of the novel ligands was performed. Most of the newly synthesized compounds showed high TSPO binding affinity levels, with *Ki* values in the picomolar to nanomolar range. The compound **GMA 15** represents the most potent TSPO binding affinity compared to the reference standards DPA-714 and PK11195. MD studies of the top molecule, **GMA 15**, were carried out to determine its time-dependent stability with the receptor compared to DPA-714 and PK11195. They indicated that the compound **GMA 15** might have a better interaction profile and better potency than both PK11195 and the core scaffold DPA-714. The results of the study suggested that novel *N*,*N*-disubstituted pyrazolopyrimidine acetamides may represent a promising class of compound, and **GMA 15** may serve as a novel TSPO ligand suitable for potential therapeutic applications.

## Figures and Tables

**Table 1:** In vitro binding affinity and lipophilicity of new pyrazolopyrimidine derivatives.

Compound	R_1_	R_2_	*Ki* (nM) ^1^	cLogP ^2^
**GMA 7**	-Me	-CH_2_CH_3_	17.10	3.83
**GMA 8**	-CH_2_CH_3_	-CH_2_CH_3_	8.79	4.36
**GMA 9**	-CH_2_CH_3_	-CH(CH3)_2_	13.27	4.66
**GMA 10**	-CH_2_ CH_2_CH_3_	-CH_2_CH_2_CH_3_	0.18	5.41
**GMA 11**	-CH_2_CH_3_	-CH_2_CH_2_OCH_3_	0.19	4.34
**GMA 12**	-CH_2_CH_2_OCH_3_	-CH_2_CH_2_OCH_3_	25.37	4.32
**GMA 13**	-CH_2_CCH	-CH_2_CCH	0.90	4.50
**GMA 14**	-CH_3_	-C_6_H_5_	N.D. ^3^	5.21
**GMA 15**	-CH_2_CH_3_	-C_6_H_5_	0.06	5.74
**GMA 16**	-CH_2_CH_3_	-CH_2_-2-OMe-C_6_H_4_	0.49	5.43
**GMA 17**	-CH_2_CH_3_	-CH_2_-2,5-OMe-C_6_H_4_	0.54	5.52
**PK11195**			1.34	4.6
**DPA-714**			3.66	3.33

^1^ Affinity of compounds determined by displacement of [^3^H]PK11195 from isolated human leukocytes. ^2^ The c log P was calculated with ACD/ChemSketch software. ^3^ N.D. = not determined for insolubility. The data are expressed as the mean values, and SD values are not displayed in the table.

**Table 2:** Glide XP docking results of **GMA 10**, **GMA 11**, **GMA 15**, PK11195 and DPA-714 with five binding sites of TSPO.

Compound	Total Score	Amino Acid Involved in Active Pocket in 3 Å	Involved Group of Amino Acid	Length of H-Bond Å	No. of H-Bond
**GMA 10**	−9.206	Phe-55, Cys-107, Val-110, Ile-47, Trp-51, Pro-42, Phe-141, Ala-142, Ile-145, Ser-146, Ile-149, Ile-27, Tyr-32, Ser-91, Phe-90, Ser-21, Ser-22, Asn-87	Trp-51	2.6	1
**GMA 11**	−8.988	Phe-55, Cys-107, Val-110, Ile-47, Trp-51, Pro-42, Phe-141, Ala-142, Ile-145, Ser-146, Ile-149, Ile-27, Tyr-32, Ser-91, Phe-90, Ser-21, Ser-22, Asn-87, Phe-95, Tyr-32, Gln-94, Tyr-135, Trp-138	Trp-138 Trp-51	2.5 2.2	2
**GMA 15**	−12.443	Phe-55, Asn-87, Ser-22, Ser-21, Phe-90, Ser-91, Cys-107, Val-110, Tyr-135, Trp-135, Trp-138, Ser-139, Pro-42, Ile-47, Phe-141, Ala-142, Ile-145, Leu-145, Ser-146, Ile-149, Trp-51, Ile-27, Tyr-32	Trp-138 Trp-51	2.5 2.1	2
**PK11195**	−10.083	Phe-55, Ser-22, Val-110, Asn-87, Cys-107, Ile-47, Phe-90, Ser-91, Trp-138, Trp-51, Phe-141, Pro-42, Ala-142, Leu-145	Trp-138 Trp-51	2.7 2.1	2
**DPA-714**	−8.252	Ser-21, Ser-91, Asn-87, Phe-90, Cys-107, Val-110, Ser-146, Leu-145, Ala-142, Phe-142, Phe-141, Trp-138, Pro-42, Tyr-32, Gly-44, Ile-27, Ile-47	Trp-138 Trp-51	2.4 2.0	2

## Data Availability

Data is contained within the article and supplementary material.

## Supplementary Materials

The following supporting information can be downloaded at: https://www.mdpi.com/article/10.3390/ph16040576/s1, Table S1: In silico physicochemical and ADMET studies; Table S2: Cytotoxicity data; Figure S1: Cytotoxicity data; Figure S2: Molecular Docking Studies; Figures S3–S24: ^1^H-NMR and ^13^C-NMR spectra for **GMA 7**–**17**.
