# Supplementary material for: Synthesis, In Silico and In Vitro Characterization of Novel N,N-Substituted Pyrazolopyrimidine Acetamide Derivatives for the 18KDa Translocator Protein (TSPO)
Source: Pharmaceuticals (Basel). 2023 Apr 11;16(4):576. doi: 10.3390/ph16040576 (PMC10142799; doi:10.3390/ph16040576)
Supplement: Supplementary file 1 [file pharmaceuticals-16-00576-s001.zip › pharmaceuticals-2225577-supplementary-update.pdf]

Supplementary Material

Synthesis, In Silico and in vitro characterization of novel *N,N*-substituted pyrazolopyrimidine acetamide derivatives for the 18KDa translocator Protein (TSPO)

Jaekyung Park [1], Sobia Wasim [2], Jae Ho Jung [3], Mi-hyun Kim [4], Byung Chul Lee [3,5], Mohammad Maqusood Alam [6,*] and Sang-Yoon Lee [1,6,7,*]

[1]Gachon Advanced Institute for Health Science and Technology, Graduate School, Gachon University, Incheon 21999, Republic of Korea

[2]Department of Biochemistry, College of Medicine, Gachon University, Incheon 21999, Republic of Korea

[3]Department of Nuclear Medicine, Seoul National University College of Medicine, Seoul National University Bundang Hospital, Seongnam 13620, Korea

[4]Gachon Institute of Pharmaceutical Science and Department of Pharmacy, College of Pharmacy, Gachon University, Incheon 21936, Republic of Korea

[5]Center for Nanomolecular Imaging and Innovative Drug Development, Advanced Institutes of Convergence Technology, Suwon 16229, Korea

[6]Neuroscience Research Institute, Gachon University, Incheon 20565 South Korea

[7]Department of Neuroscience, College of Medicine, Gachon University, Incheon 21936, Korea

*Corresponding author: Prof. Dr. Sang-Yoon Lee, Tel.: +82-32-822-5360, E-mail: rchemist@gachon.ac.kr and Dr. Mohammad Maqusood Alam, Tel.: +82-32-822-9529, E-mail: maqusood.in@gmail.com.

Table of contents

Content	Page No.
**Table S1.** *In silico* physicochemical and ADMET studies	S2
**Table S2.** Cytotoxicity data for **GMA 10, GMA 11, GMA 15, DPA-714, PK11195**	S3
**Figure S1.** Cytotoxicity data for **GMA 10, GMA 11, GMA 15, DPA-714, PK11195**	S3
**Figure S2.** Molecular Docking Studies for **GMA 10, GMA 11, GMA 15, DPA-714, PK11195**	S4
**Figure S3 & S4.** ^{1}H-NMR and ^{13}C-NMR spectra for **GMA 7**	S7, S8
**Figure S5 & S6.** ^{1}H-NMR and ^{13}C-NMR spectra for **GMA 8**	S9, S10
**Figure S7 & S8.** ^{1}H-NMR and ^{13}C-NMR spectra for **GMA 9**	S11, S12
**Figure S9 & S10.** ^{1}H-NMR and ^{13}C-NMR spectra for **GMA 10**	S13, S14
**Figure S11 & S12.** ^{1}H-NMR and ^{13}C-NMR spectra for **GMA 11**	S15, S16
**Figure S13 & S14.** ^{1}H-NMR and ^{13}C-NMR spectra for **GMA 12**	S17, S18
**Figure S15 & S16.** ^{1}H-NMR and ^{13}C-NMR spectra for **GMA 13**	S19, S20
**Figure S17 & S18.** ^{1}H-NMR and ^{13}C-NMR spectra for **GMA 14**	S21, S22
**Figure S19 & S20.** ^{1}H-NMR and ^{13}C-NMR spectra for **GMA 15**	S23, S24
**Figure S21 & S22.** ^{1}H-NMR and ^{13}C-NMR spectra for **GMA 16**	S25, S26
**Figure S23 & S24.** ^{1}H-NMR and ^{13}C-NMR spectra for **GMA 17**	S27, S28

Table S1. Physiological properties and ADMET profile of the lead compound **GMA 15**.

**Physicochemical properties**		
**Parameter/Model**	**Result**	**Probability**
Molecular formula & weight	C26H27FN4O & 430.52 g/mol	
Fraction Csp3 & Num. rotatable bonds	0.27 & 8	
Num. of H-bond acceptors & donors	4 &0	-
Molar refractivity & TPSA	126.44 & 50.50 Å²	-
Log *P*o/w (MLogP)	4.53	-
Solubility & solubility class	1.03e-09 mol/l, Poorly soluble	-
**Absorption**		
Blood-brain barrier	Yes (BBB+)	0.9847
Human intestinal absorption	Yes (HIA+)	1.0000
Caco-2 permeability	No (Caco-2-)	0.5230
P-glycoprotein substrate	Yes	0.5151
P-glycoprotein inhibitor	Yes	0.9645
**Distribution**		
Subcellular localization	Mitochondria	0.8005
**Metabolism**		
CYP450 substrate	3A4	-
CYP450 non-substrate	2C9 & 2D6	-
CYP450 substrate	2C9 & 2C19	
CYP450 non-inhibitor	1A2, 2D6, & 3A4	-
CYP inhibitory promiscuity	High	0.8810
**Toxicity**		
hERG inhibition	Weak inhibitor	0.9518
AMES toxicity	Non AMES toxic	0.6277
Carcinogens	Non-carcinogens	0.5087
Acute oral toxicity	III (LD50 >500–5000 mg/kg)	0.5287
Rat acute toxicity	LD50: 2.7559 mol/kg	-
**Druglikeness**		
Lipinski's rule of five	Yes; 1 violations:MLogP>4.15	Lipinski's rule of five
Bioavailability Score	0.55	Bioavailability Score

Table S2. Cytotoxicity data for **GMA 10, GMA 11, GMA 15, DPA-714, PK11195**

Entry	Cell viability (%, μg/mL)						
	Blank	Control†	3.125†	6.25†	12.5†	25†	50†
GMA-10	100.00±0.00	70.39±1.60	64.48±1.19	53.44±2.02	39.78±0.74	24.63±3.13	19.82±1.19
GMA-11	100.00±0.00	70.39±1.60	61.72±0.71	58.30±3.12	39.46±0.63	33.48±2.28	28.93±1.12
GMA-15	100.00±0.00	70.39±1.60	57.88±4.04	50.65±2.39	26.02±8.01	9.60±1.60	6.60±2.15
DPA-714	100.00±0.00	70.13±0.84	50.98±4.08	47.31±3.44	45.18±1.86	32.09±2.54	11.24±1.94
PK11195	100.00±0.00	70.13±0.84	57.80±5.22	49.08±0.43	48.18±0.43	38.26±4.80	32.79±3.14

† $p < 0.001$ vs blank

1.0×10^4 cells (L-929, mouse fibroblast cells) were cultured in a 96 well plate and treated with 50 μL of each concentration. After incubation at 37 ° C for 24 hours under CO_2 fluid, the treated culture medium was removed and the absorbance of each samples were measured at 570 and 650 nm using MTT reagent.

Blank was administered with only 50uL of cell incubation media. As a control, a 50 μL solution containing 1% DMSO diluted in media was administered.
MTT assay followed ISO 10993-5, ISO 10993-12 standard methods.

Figure S1. Cytotoxicity data for **GMA 10, GMA 11, GMA 15, DPA-714, PK11195**

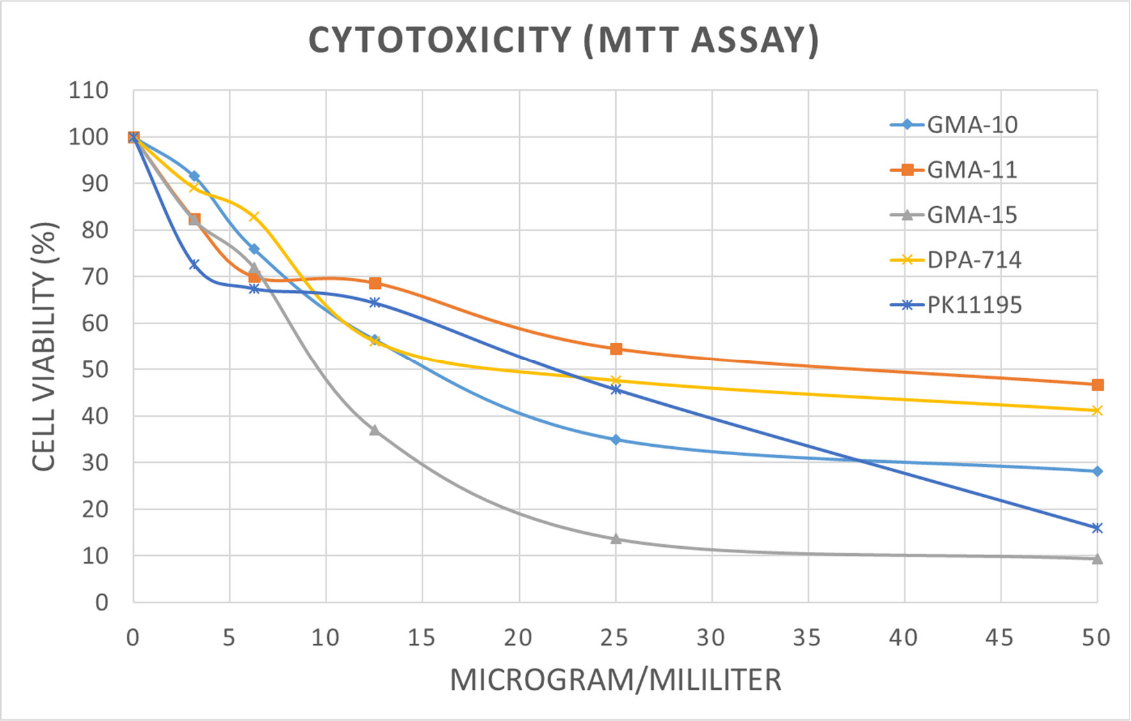

IC_{50} values of cell viability for GMA 10, GMA 11, GMA 15, DPA-714, PK11195 were 15.24, 36.22, 9.73, 18.62, 22.13 μg/mL respectively. (37.12, 87.80, 22.60, 46.73, 62.72 μM)

Figure S2. Molecular Docking Studies for **GMA 10, GMA 11, GMA 15, DPA-714, PK11195**

In order to analyze the docking properties between ligands and *Bacillus cereus* TSPO (BcTSPO), *in silico* docking tool (GLIDE) was employed and afforded the docking scores and the interactions mainly contributed for binding.

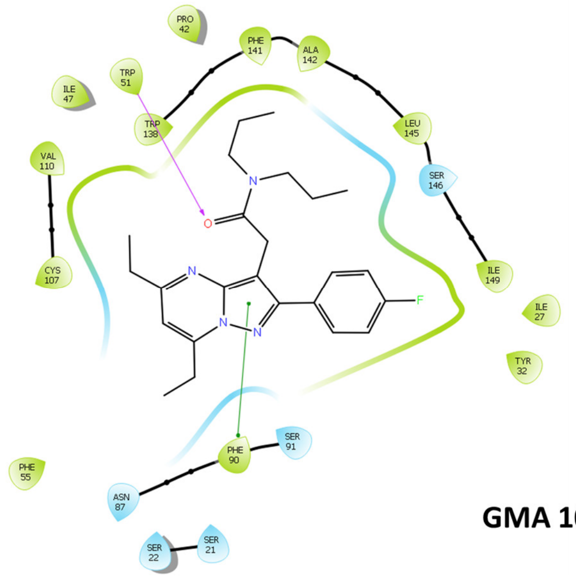

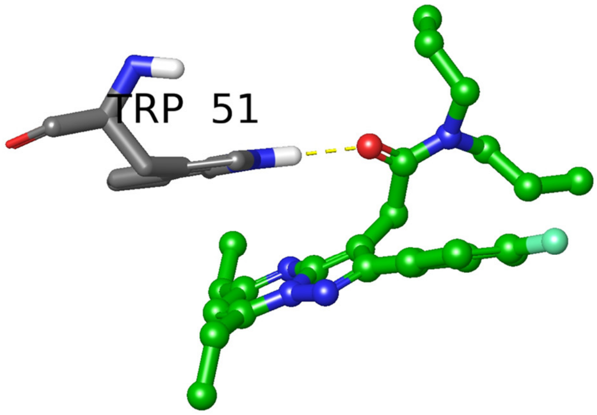

GMA 10

Docking Score: -9.206

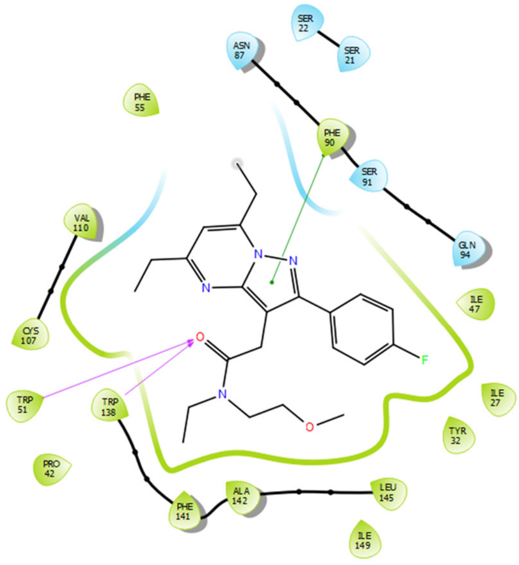

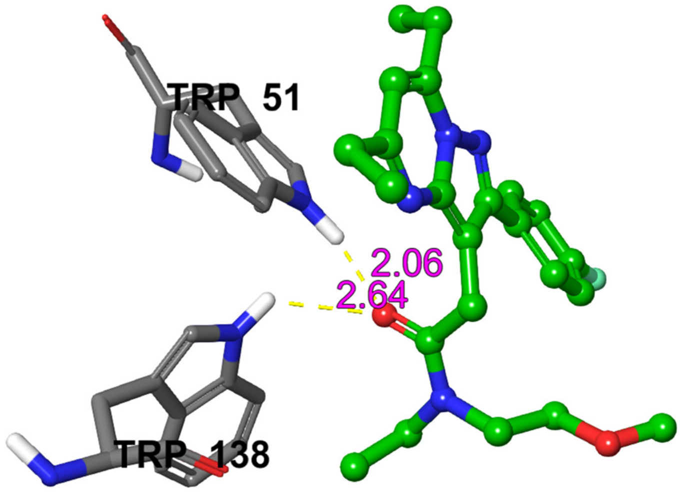

Docking Score: -9.690

GMA-11

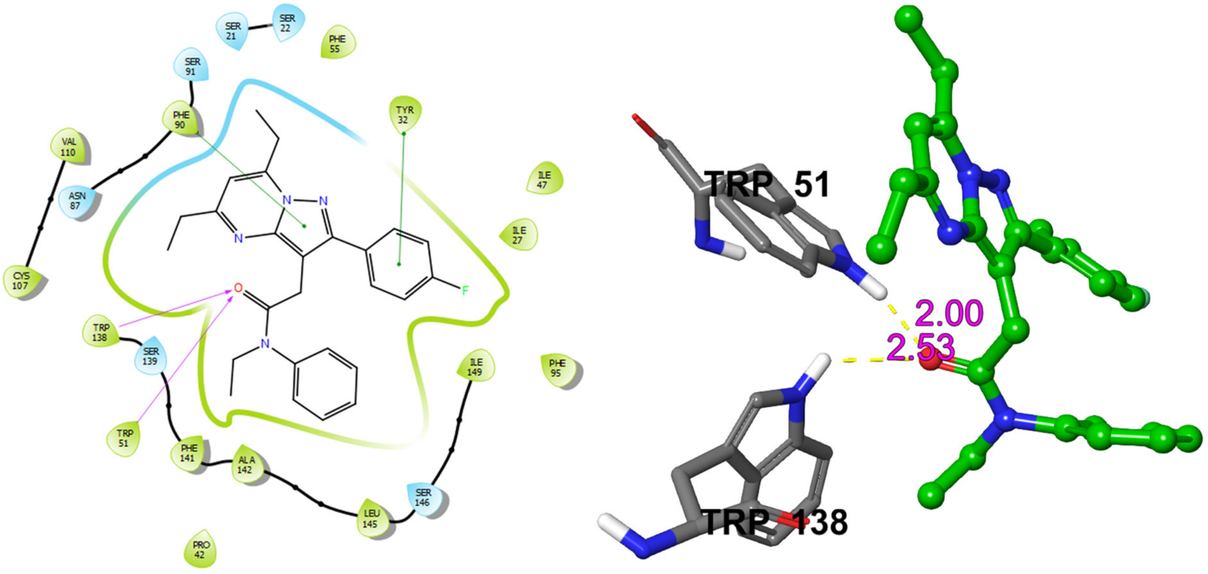

GMA 15 **Docking Score: -11.120**

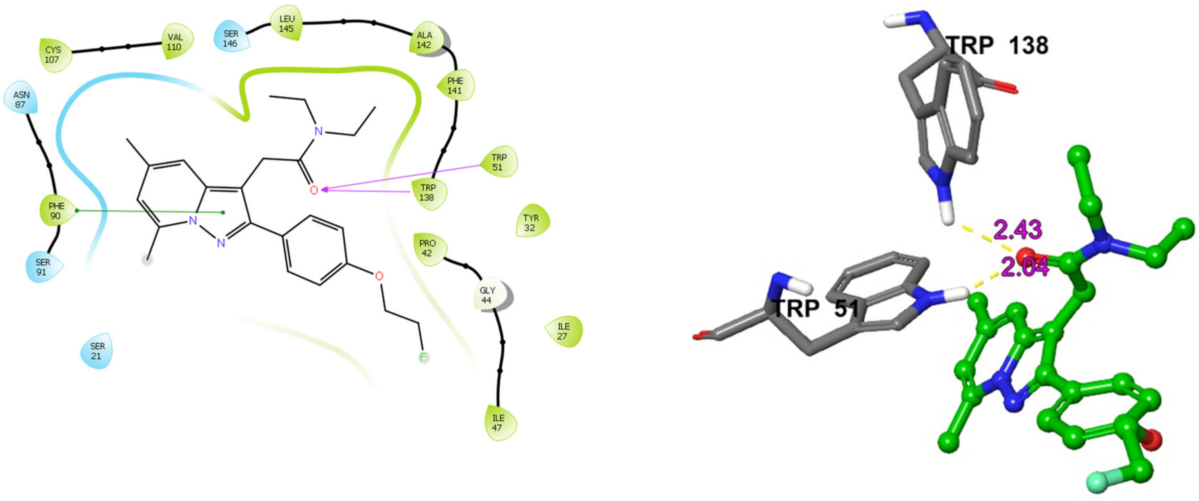

DPA-714 **Docking Score: -8.252**

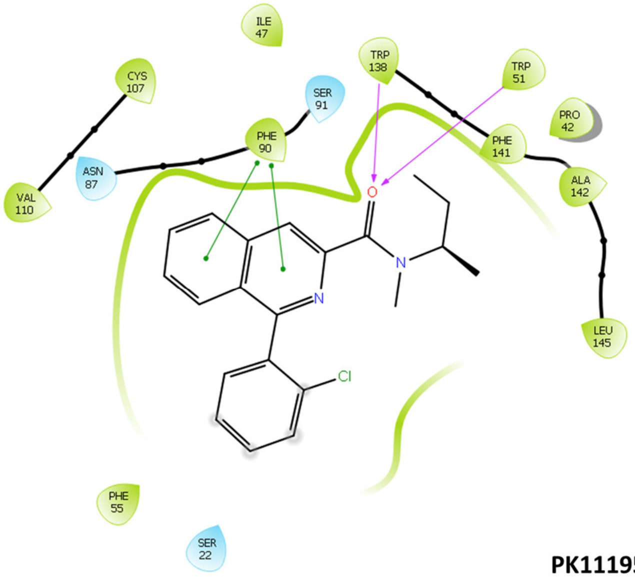

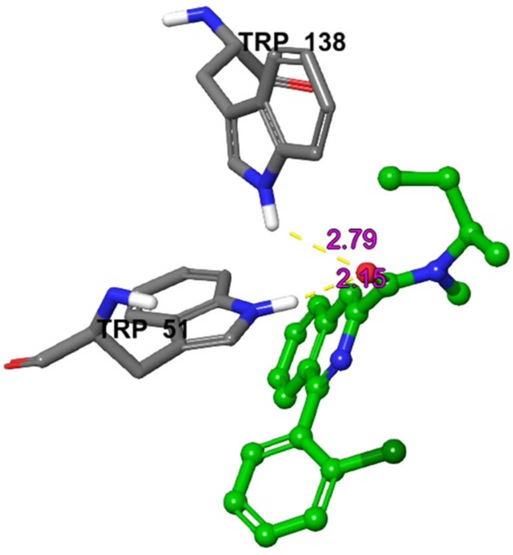

PK11195 Docking score = -10.083

Figure S3. ^{1}H-NMR spectra for **GMA 7**

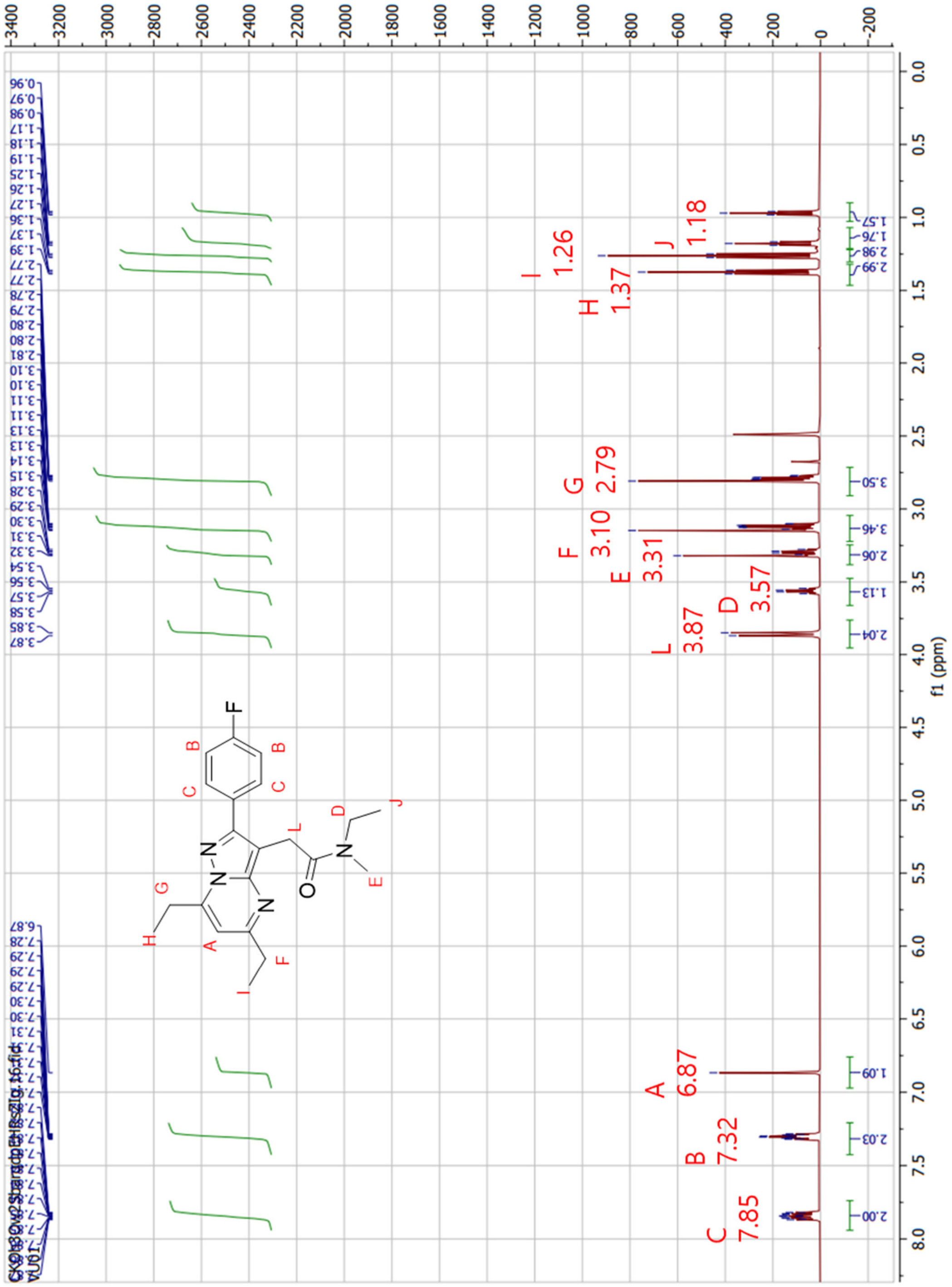

Figure S4. ^{13}C-NMR spectra for **GMA 7**

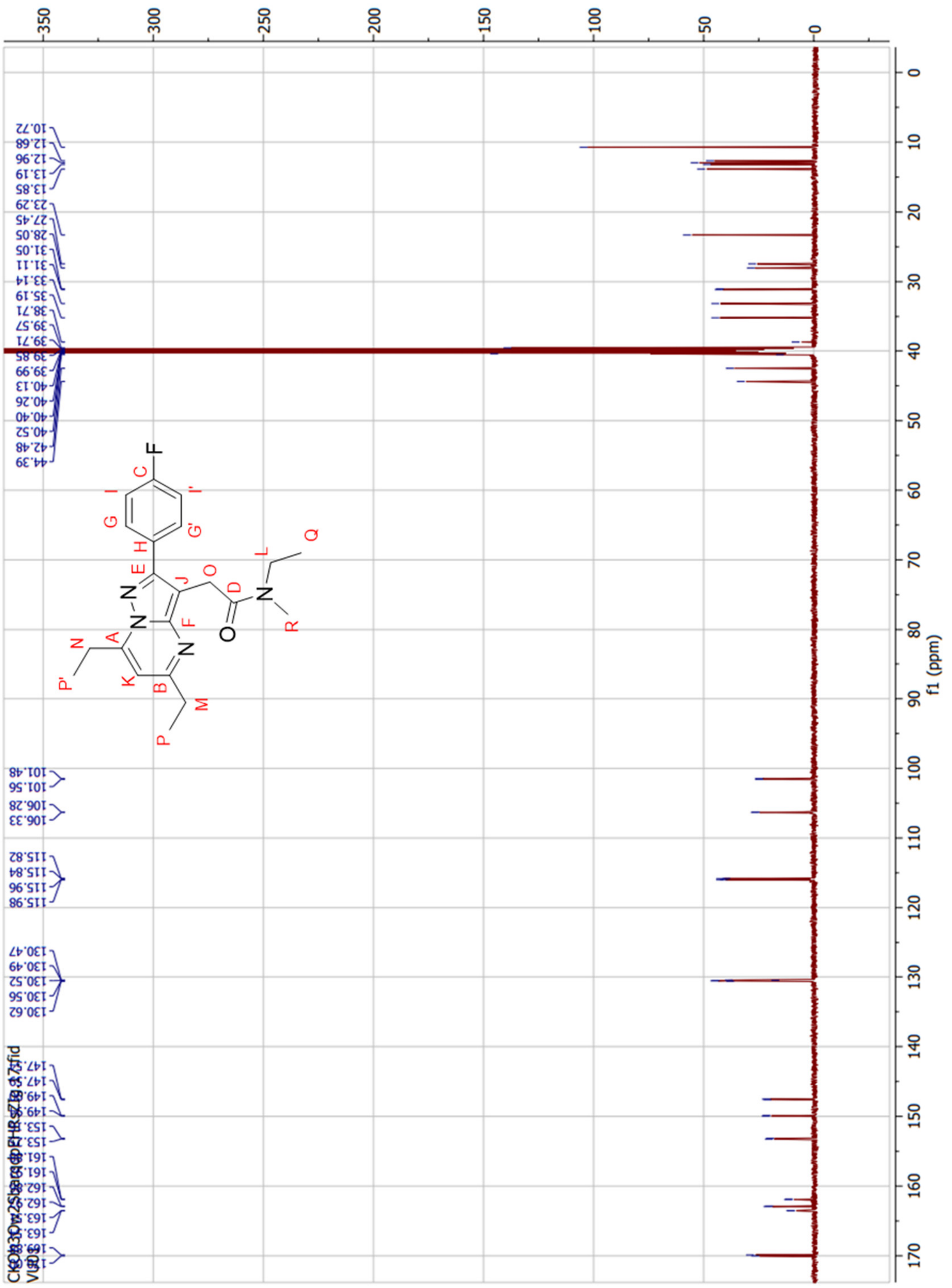

A (170.00), B (163.54), C (162.93), D (161.93), E (153.24), F (149.94), GG’ (147.59), H (130.52), II’ (115.98), J (106.33), K (101.56), L (40.13), M (31.11), N (28.05), O (23.29), PP’ (13.85), Q (10.72), R (35.19)

Figure S5. ^{1}H-NMR spectra for **GMA 8**

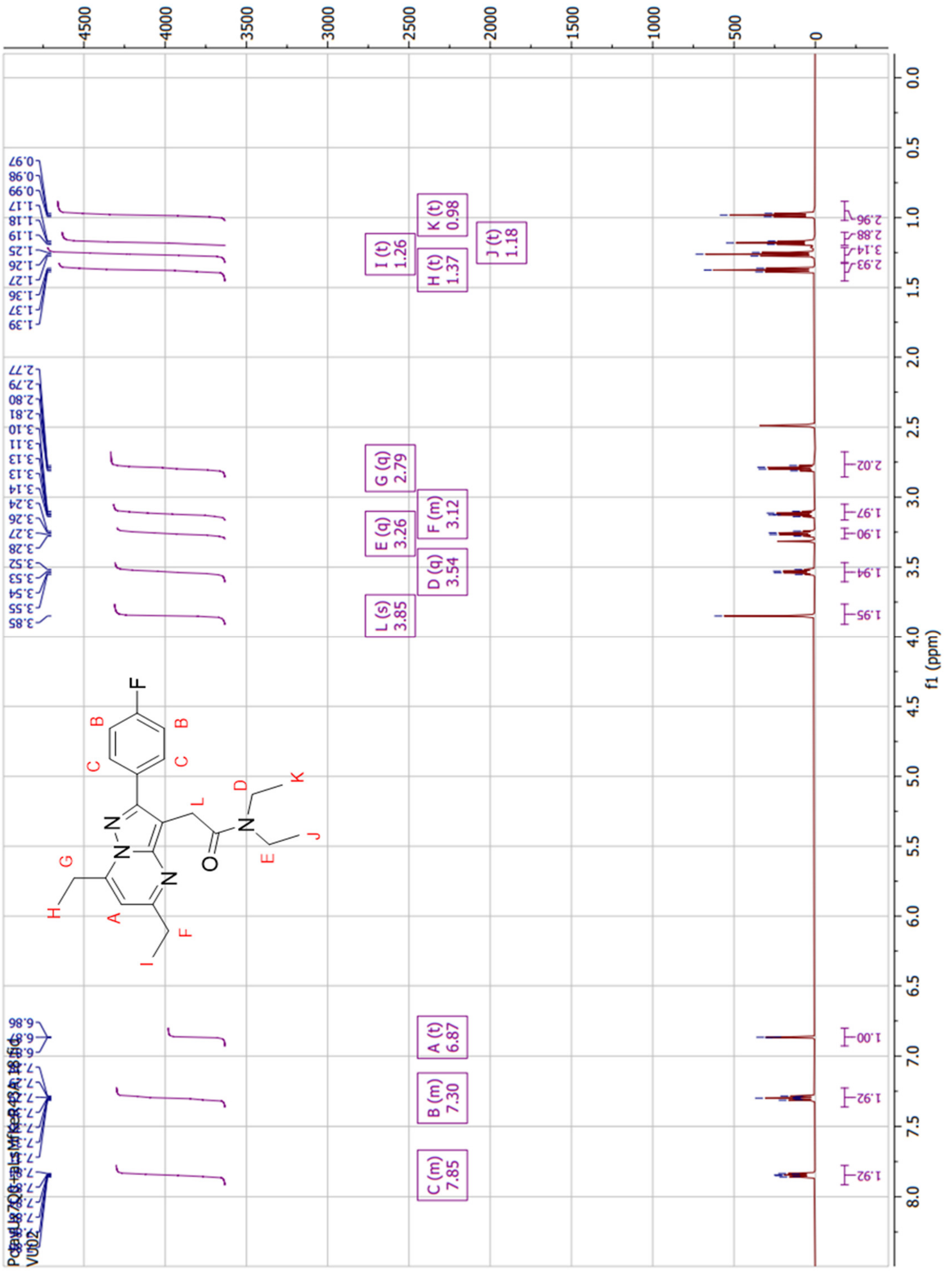

Figure S6. ^{13}C-NMR spectra for **GMA 8**

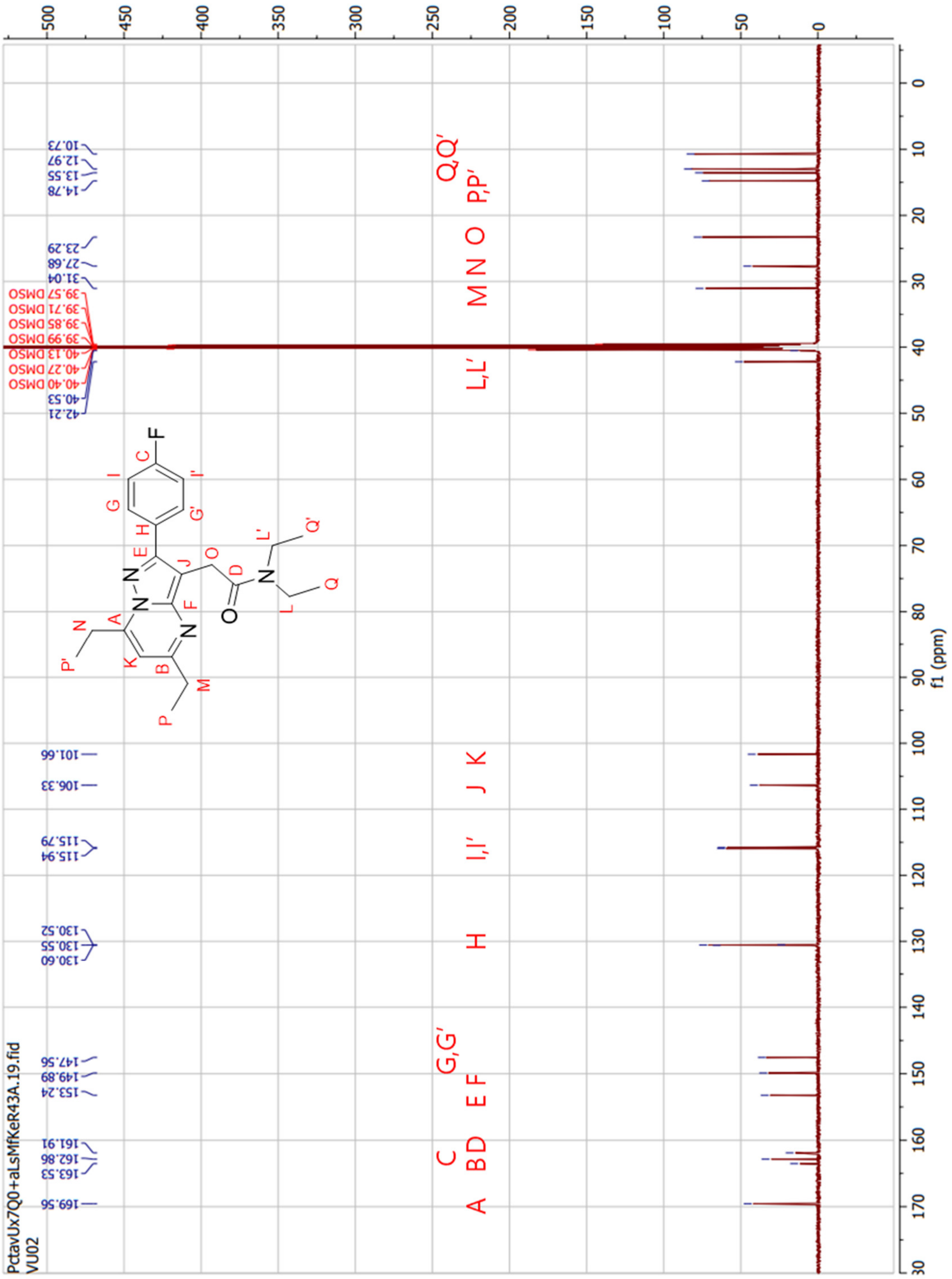

A (169.56), B (163.53), C (162.86), D (161.91), E (153.24), F (149.89), GG' (147.56), H (130.55), II' (115.94), J (106.33), K (101.66), LL' (42.21), M (31.04), N (27.68), O (23.29), PP' (14.78), QQ' (12.97)

Figure S7. ^{1}H-NMR spectra for **GMA 9**

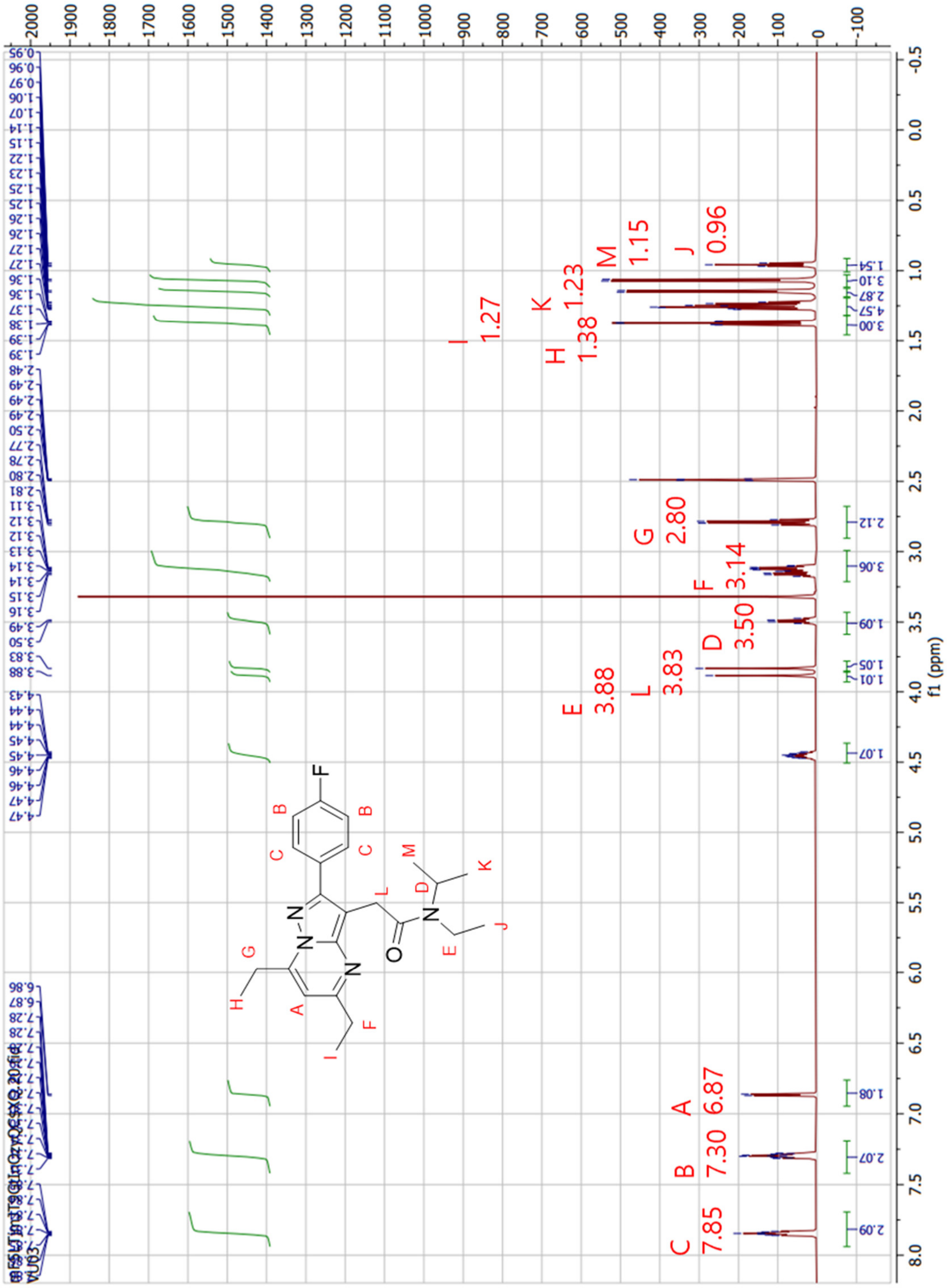

Figure S8. ^{13}C-NMR spectra for **GMA 9**

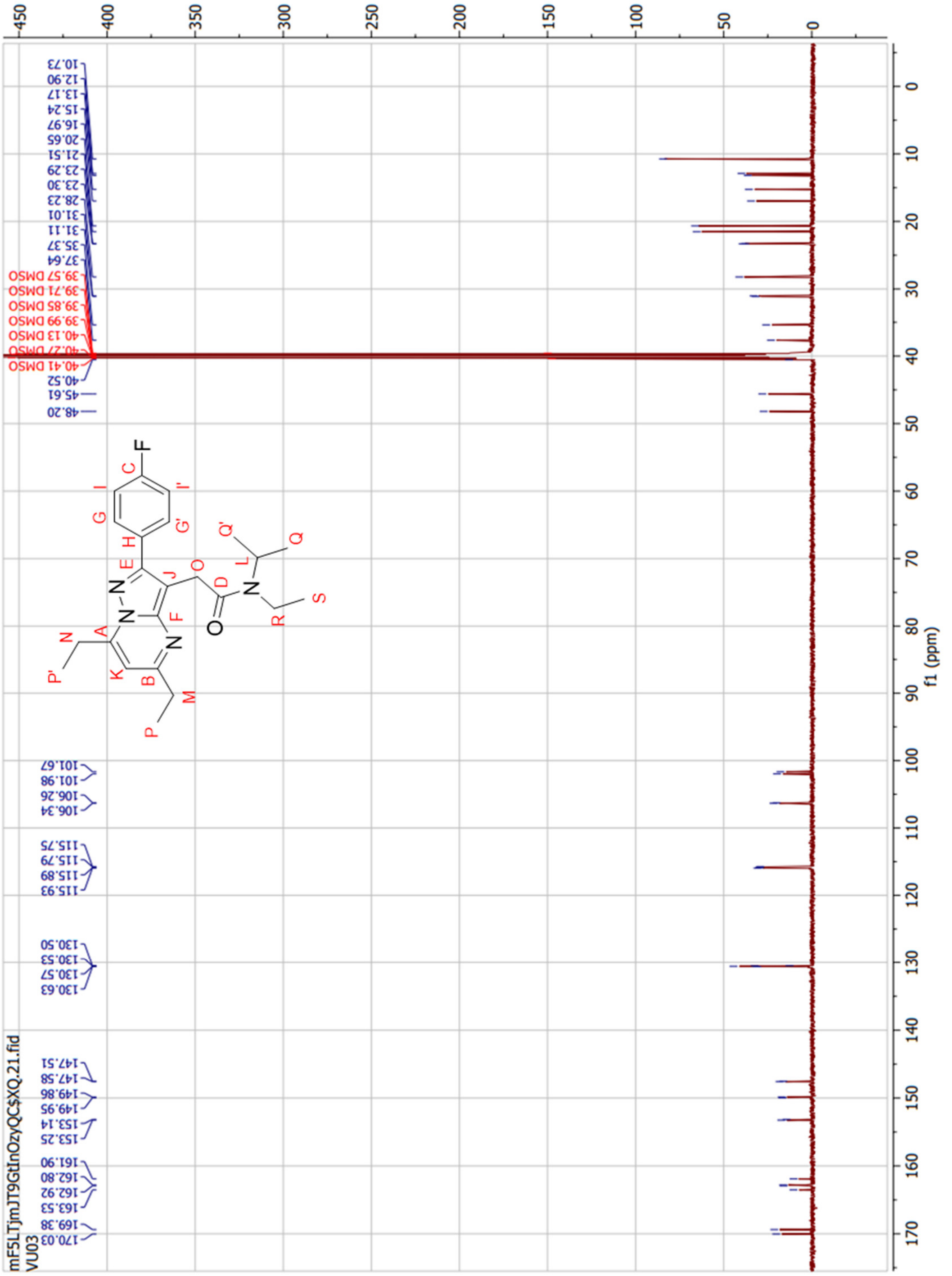

A (170.03), B (163.53), C (162.92), D (161.90), E (153.25) ,F (149.95), GG' (147.58), H (130.63), II' (115.93), J (106.34), K (101.98), L (48.20), M (31.11), N (28.23), O (23.30), PP' (13.17), QQ' (21.51), R (37.64), S (16.97)

Figure S9. ^{1}H-NMR spectra for **GMA 10**

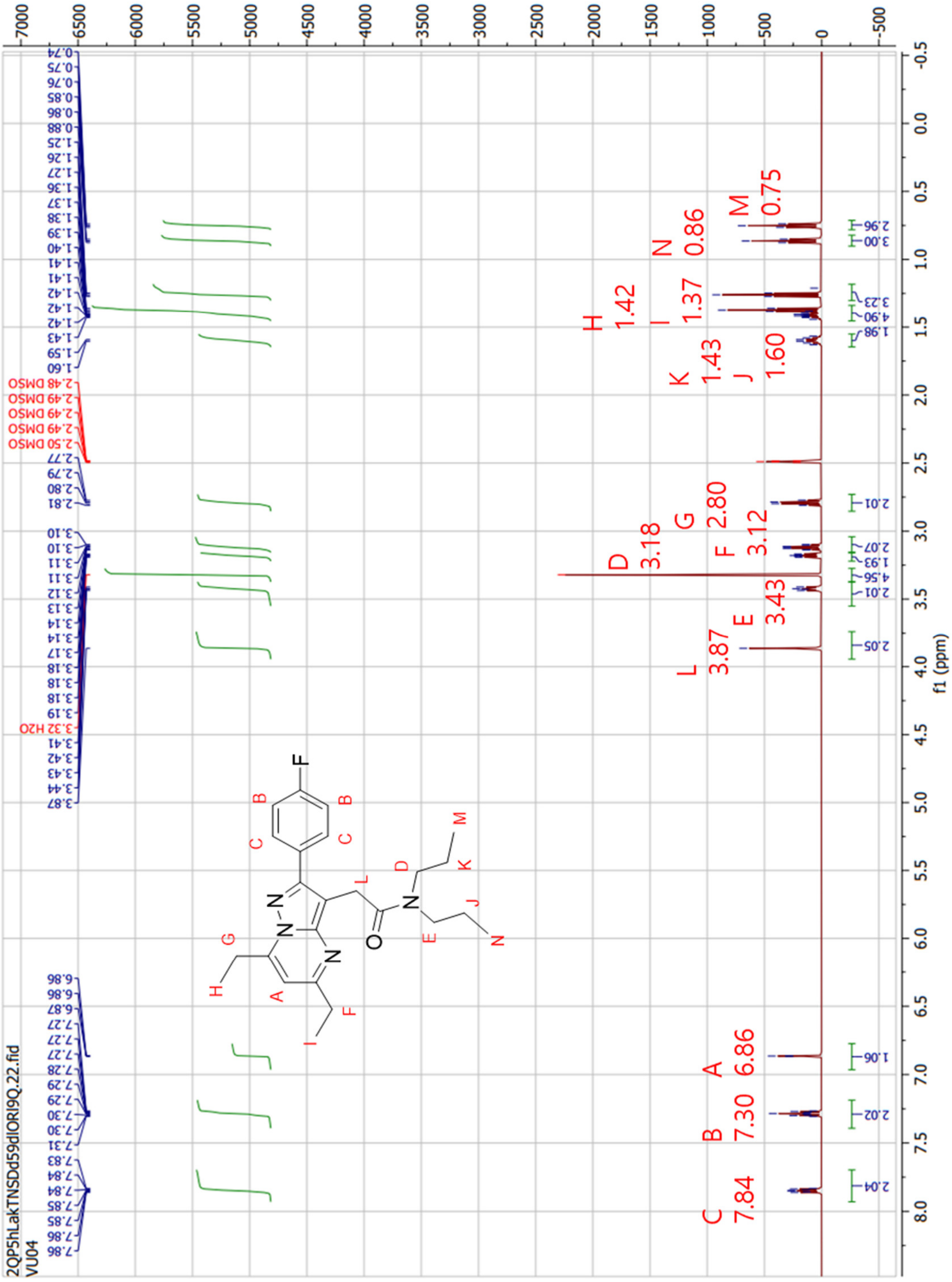

Figure S10. ^{13}C-NMR spectra for **GMA 10**

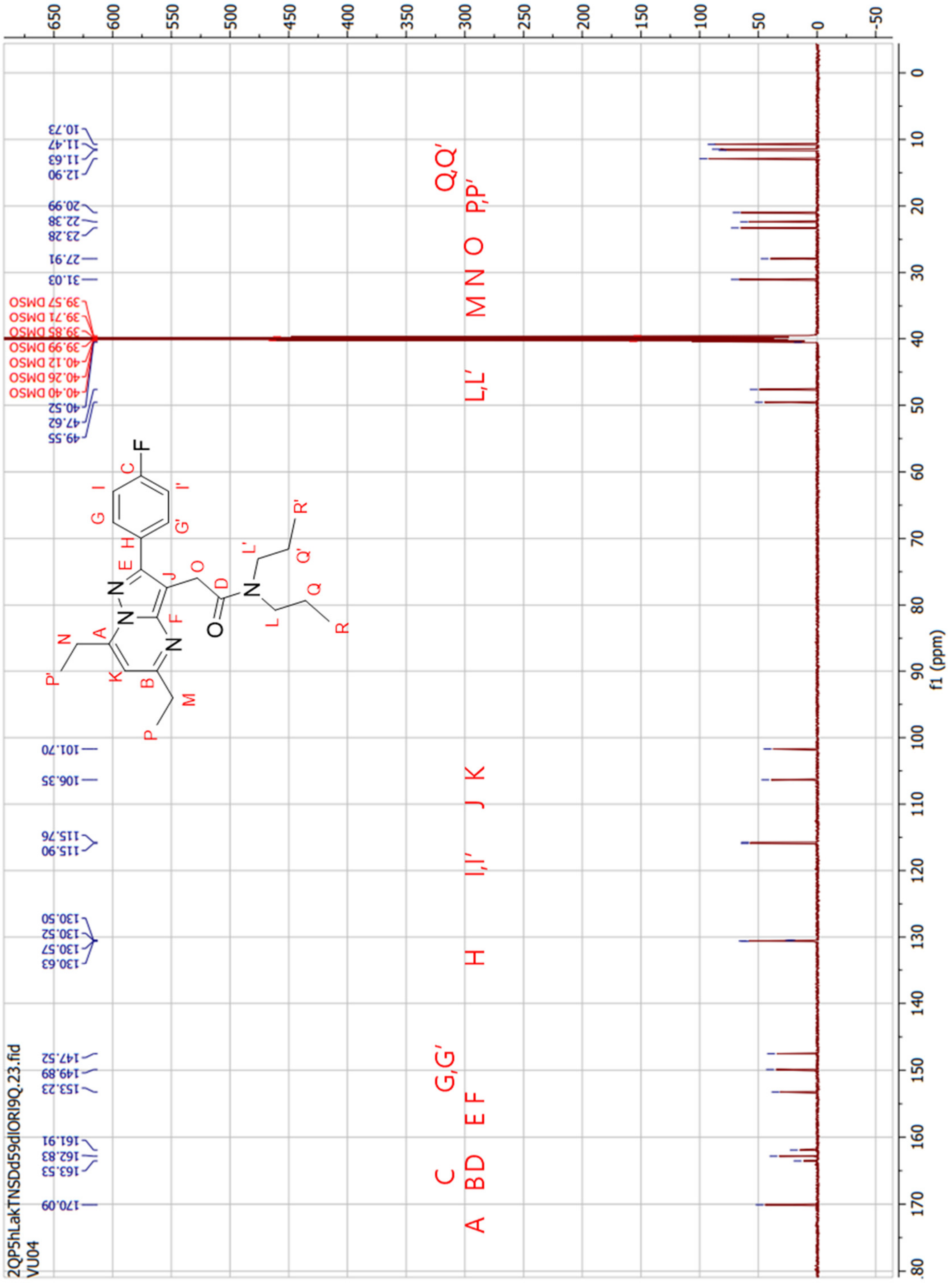

A (170.09), B (163.53), C (162.83), D (161.91), E (153.23) ,F (149.89), GG' (147.52), H (130.63), II' (115.90), J (106.35), K (101.70), LL' (49.55), M (31.03), N (27.91), O (23.28), PP' (12.90), QQ' (20.99), RR' (11.47)

Figure S11. ^{1}H-NMR spectra for **GMA 11**

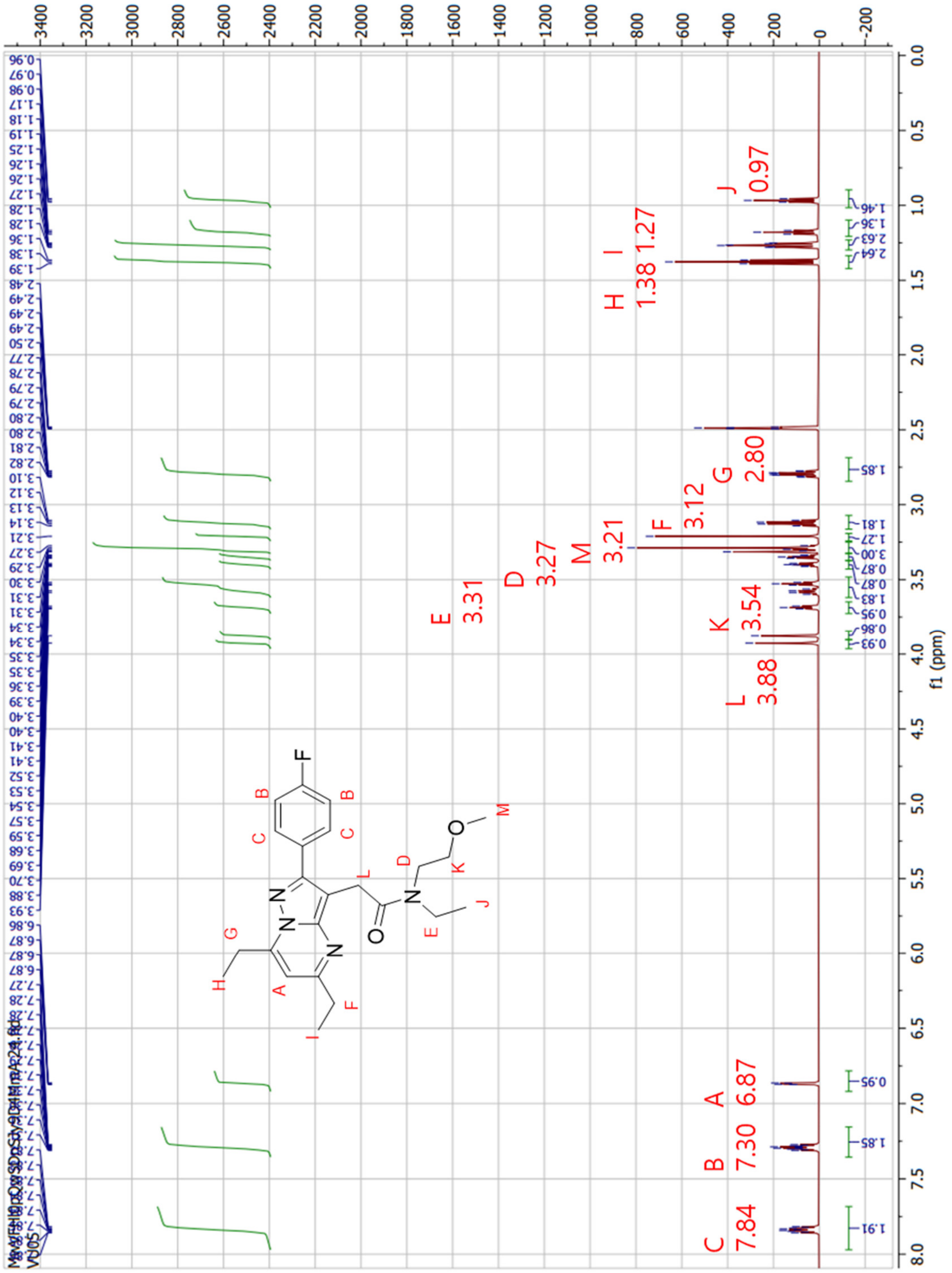

Figure S12. ^{13}C-NMR spectra for **GMA 11**

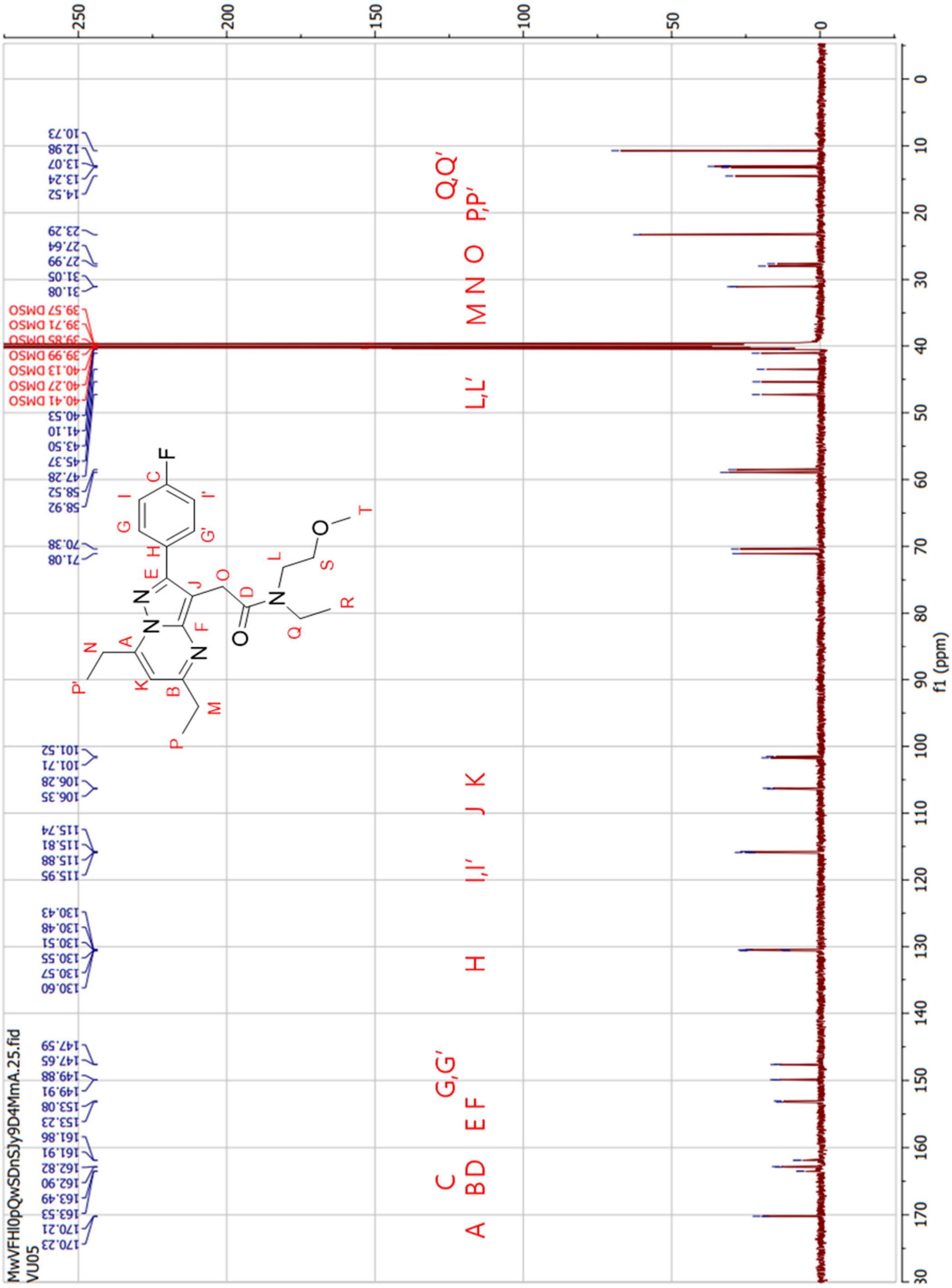

A (170.23), B (163.53), C (162.82), D (161.91), E (153.23), F (149.91), GG’ (147.65), H (130.60), II’ (115.95), J (106.35), K (101.71), L (47.28), M (31.08), N (27.99), O (23.29), PP’ (10.73), Q (41.10), R (14.52), S (71.08), T (58.92)

Figure S13. ^{1}H-NMR spectra for **GMA12**

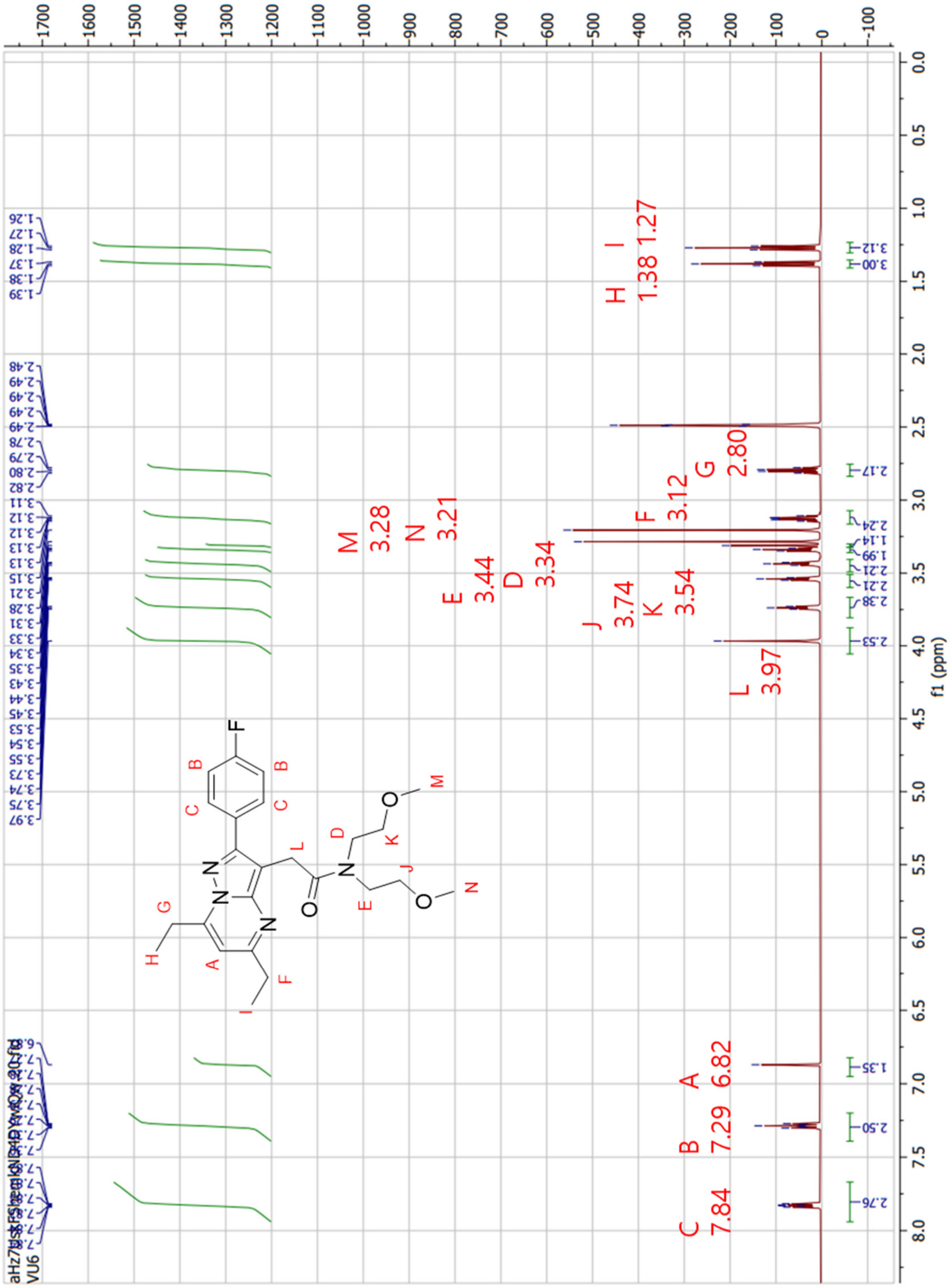

Figure S14. ^{13}C-NMR spectra for **GMA12**

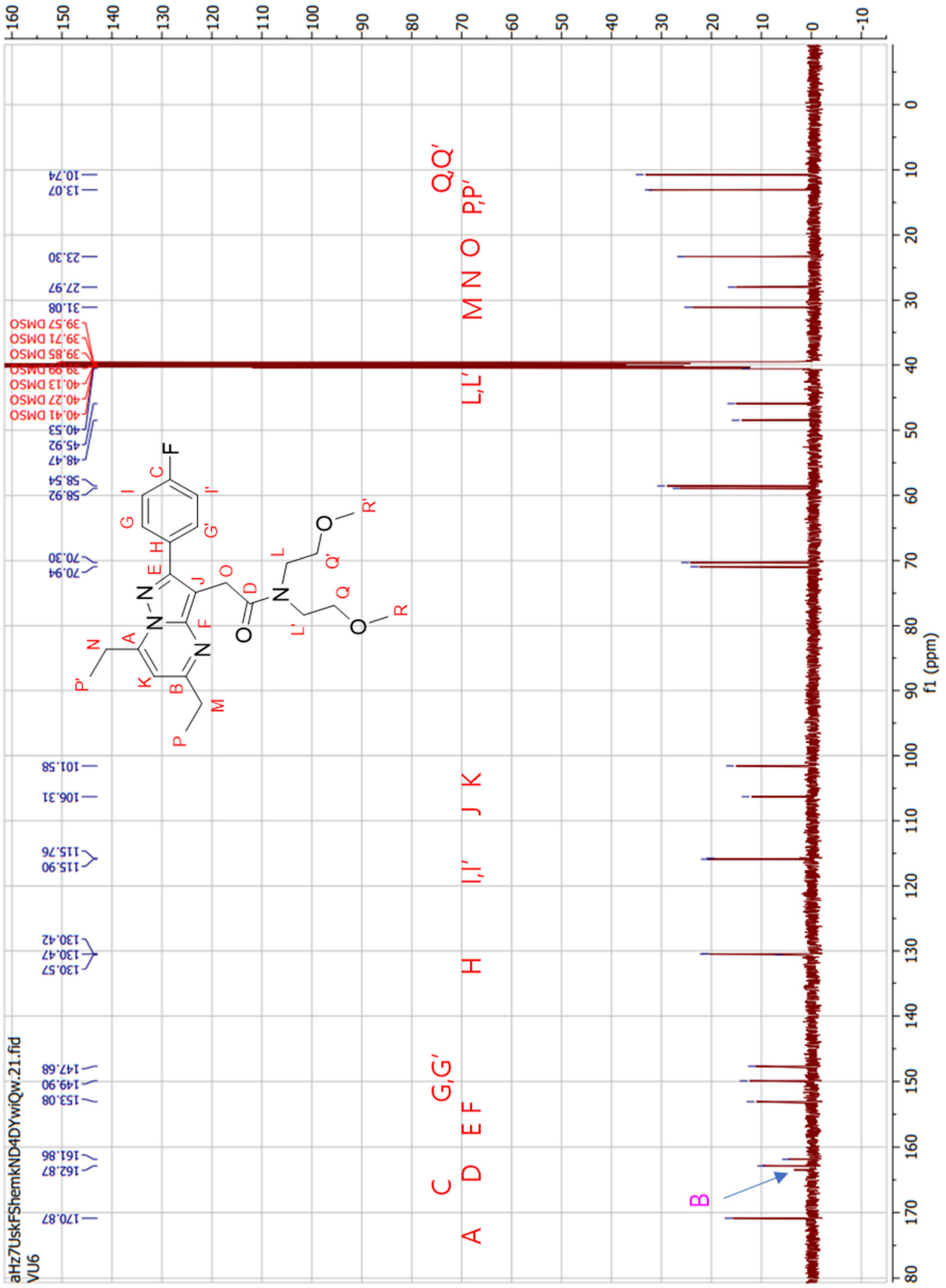

A (170.87), B (hidden), C (162.87), D (161.86), E (153.08), F (149.90), GG' (147.68), H (130.57), II' (115.90), J (106.31), K (101.58), L (48.47), M (31.08), N (27.97), O (23.30), PP' (13.07), QQ' (70.94), RR' (58.92)

Figure S15. ^{1}H-NMR spectra for **GMA13**

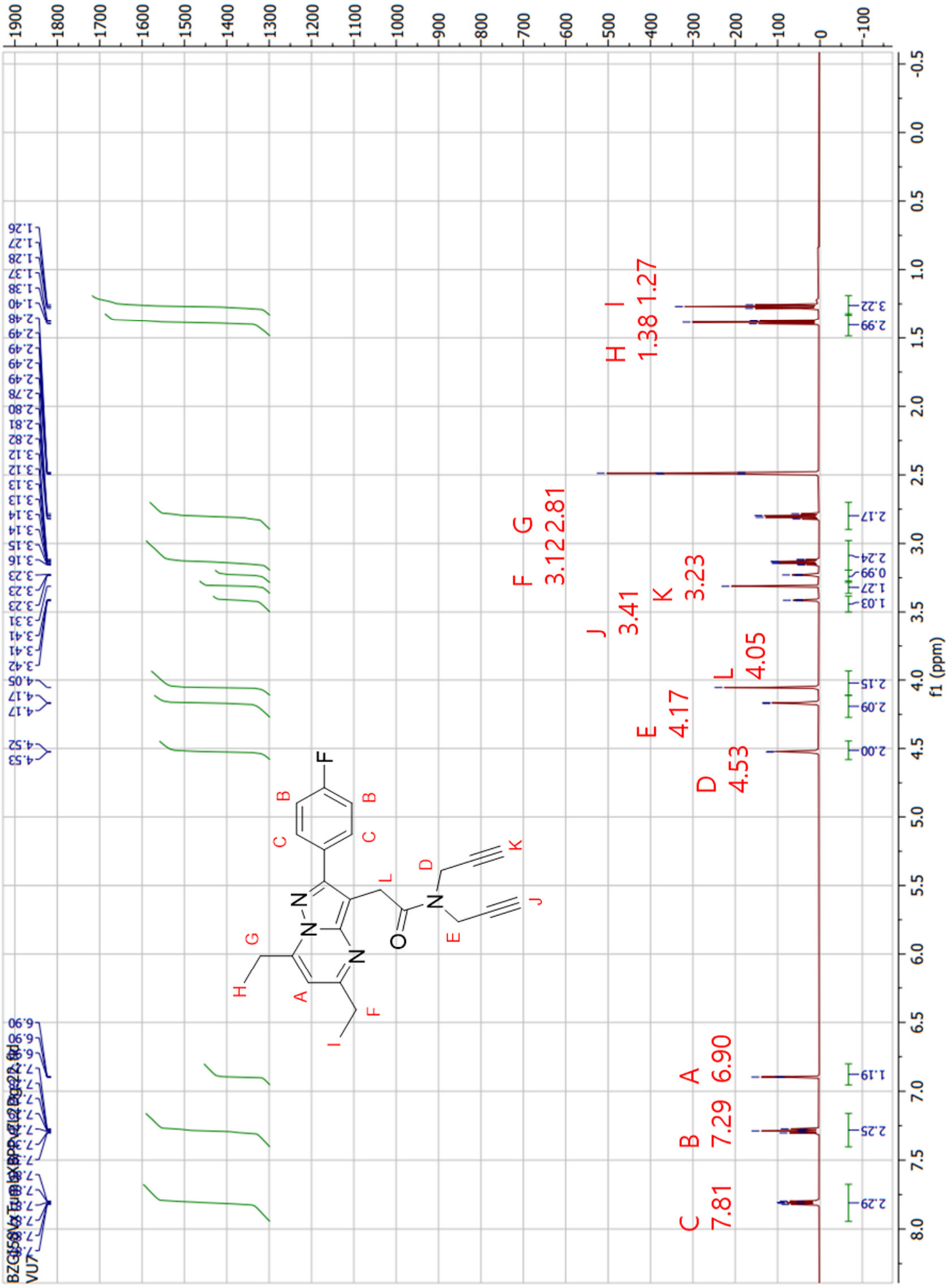

Figure S16. ^{13}C-NMR spectra for **GMA13**

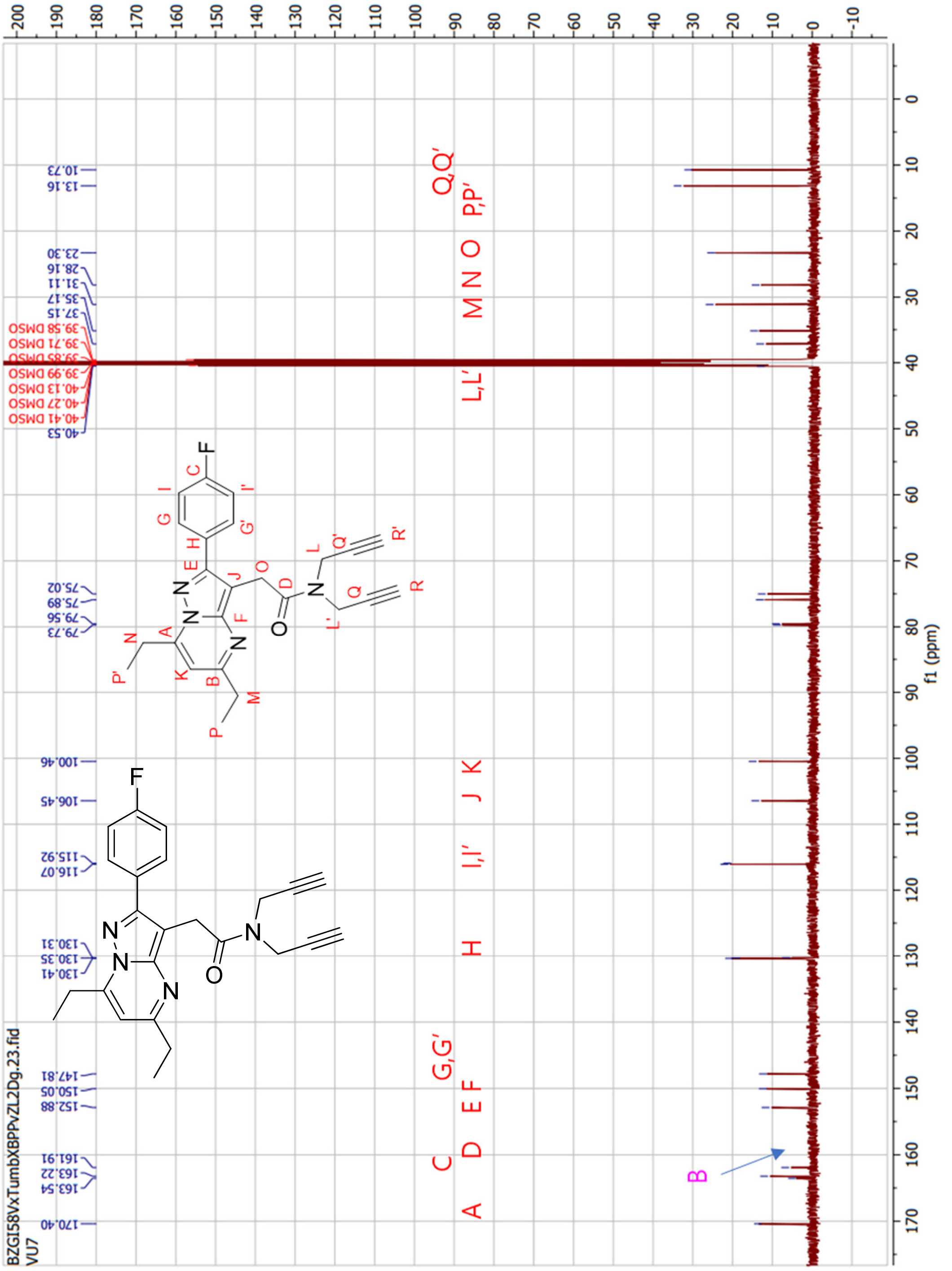

A (170.40), B (163.54), C (163.22), D (161.91), E (152.88) ,F (150.05), GG’ (147.81), H (130.35), II’ (116.07), J (106.45), K (101.46), LL’ (37.15), M (31.11), N (28.16), O (23.30), PP’ (13.16), QQ’ (79.73), RR’ (75.89)

Figure S17. ^{1}H-NMR spectra for **GMA14**

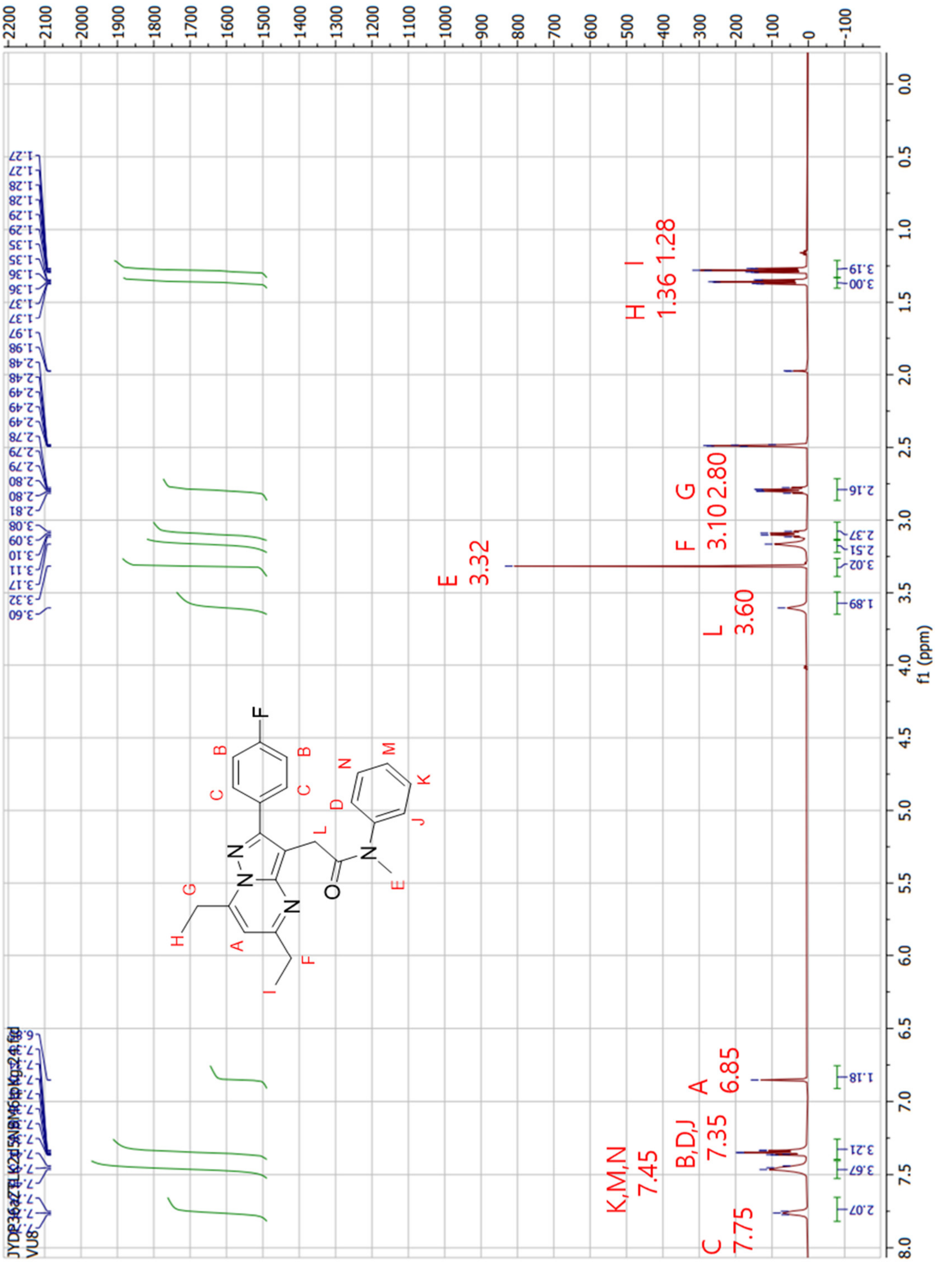

Figure S18. ^{13}C-NMR spectra for **GMA14**

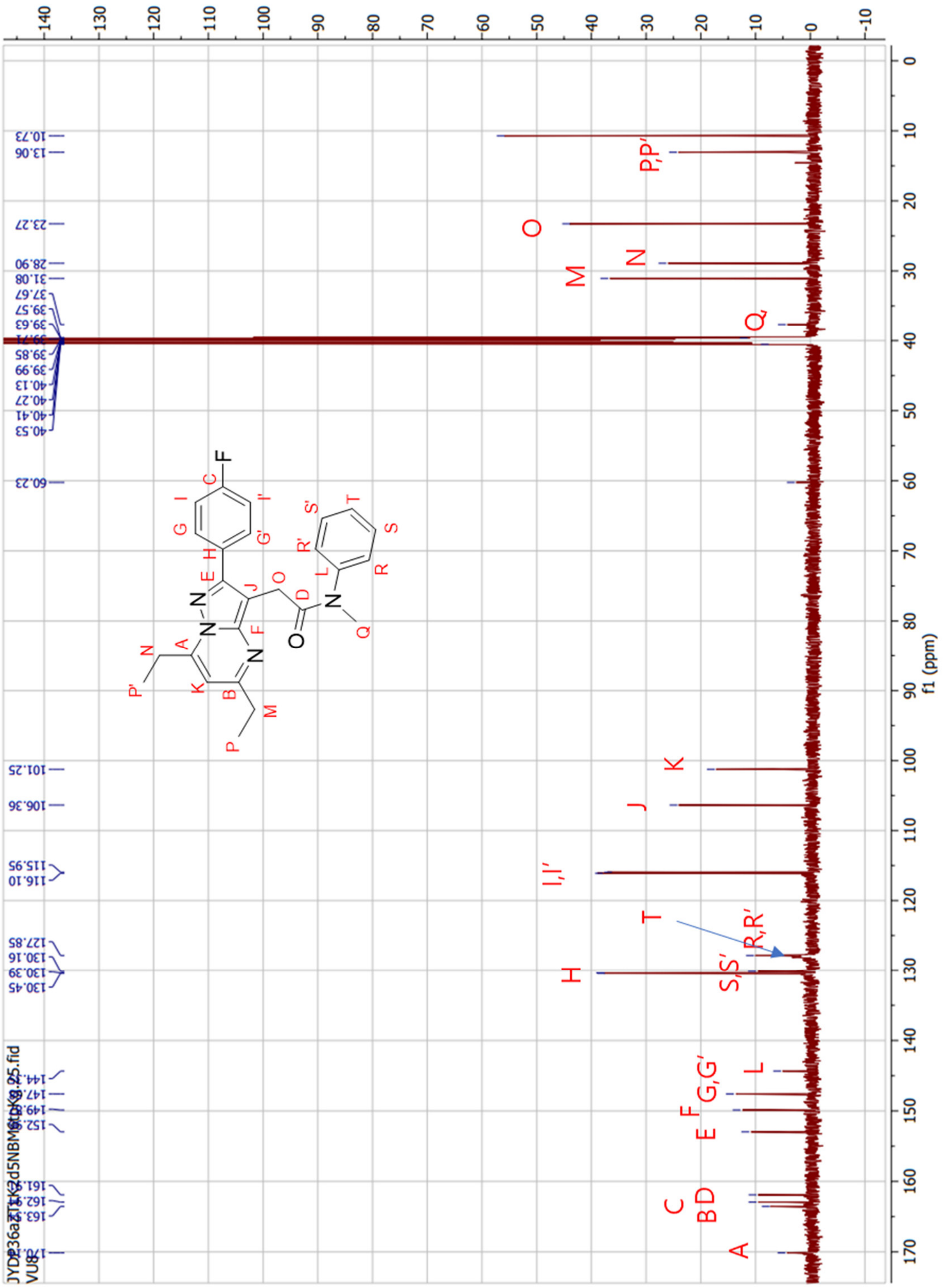

A (170.16), B (163.54), C (162.91), D (161.91), E (152.90) ,F (149.05), GG’ (147.86), H (130.39), II’ (116.10), J (106.36), K (101.25), L (144.39), M (31.08), N (28.90), O (23.27), PP’ (13.06), Q (37.67), RR’ (127.85), SS’ (130.16), T (hidden)

Figure S19. ^{1}H-NMR spectra for **GMA15**

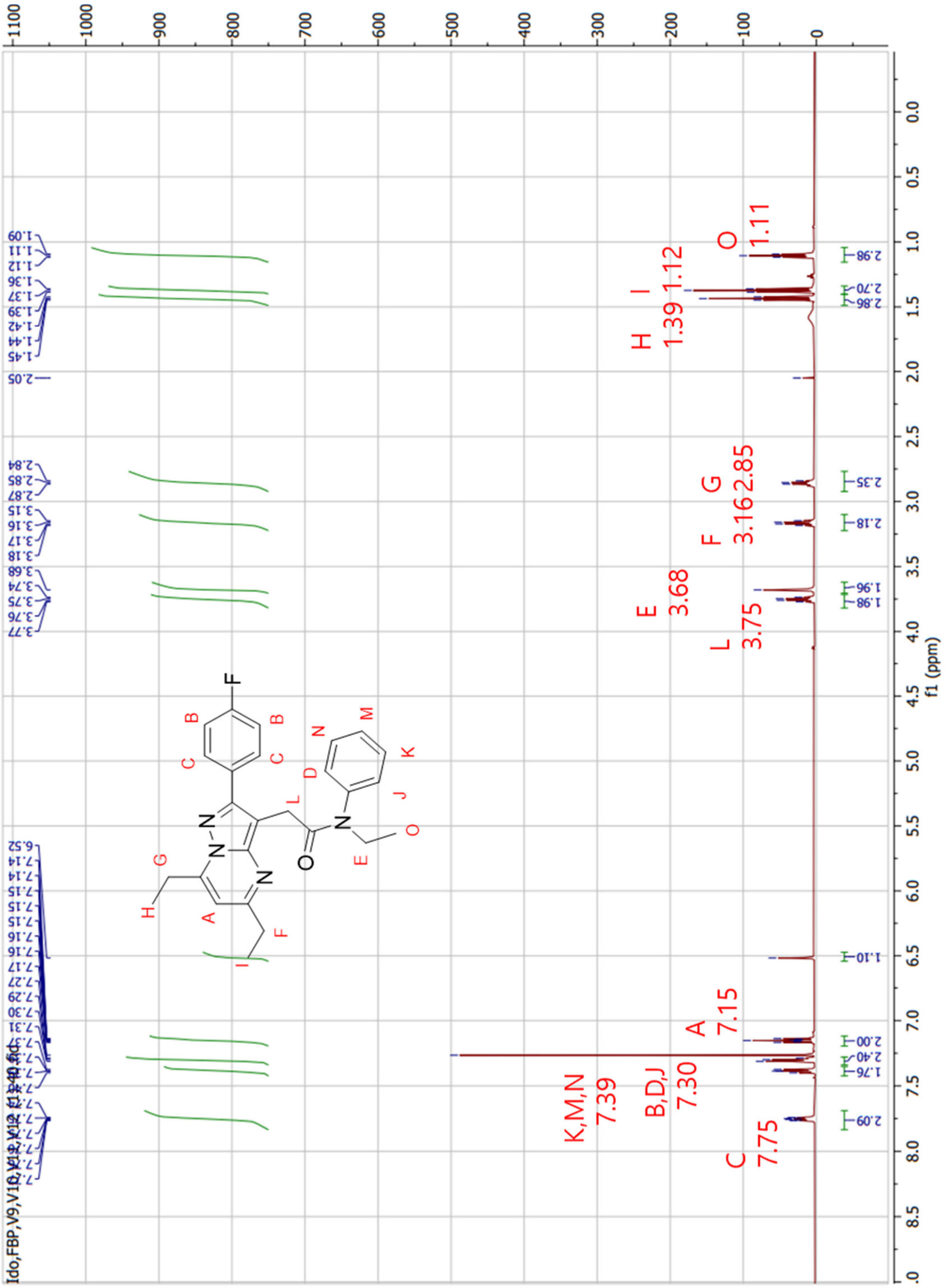

Figure S20. ^{13}C-NMR spectra for **GMA15**

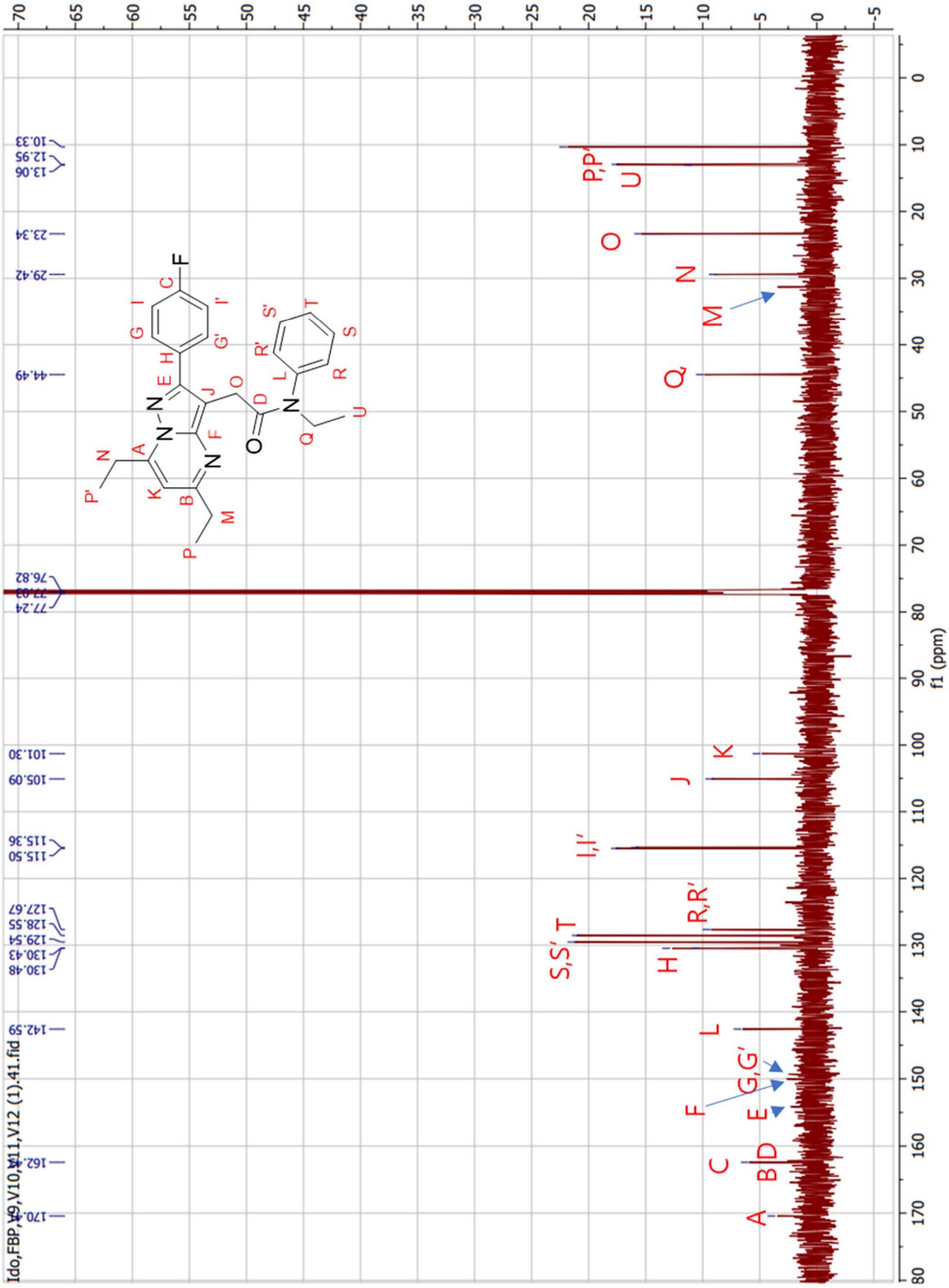

A(170.41), B(hidden), C(162.43), D(hidden), E(hidden) ,F(hidden), GG’(hidden), H(130.48), II’(115.50), J(105.09), K(101.30), L(142.59), M(hidden), N(29.42), O(23.34), PP’(12.95), Q(44.49), RR’(127.67), SS’(129.54), T(128.55), U(13.06)

Figure S21. ^{1}H-NMR spectra for **GMA16**

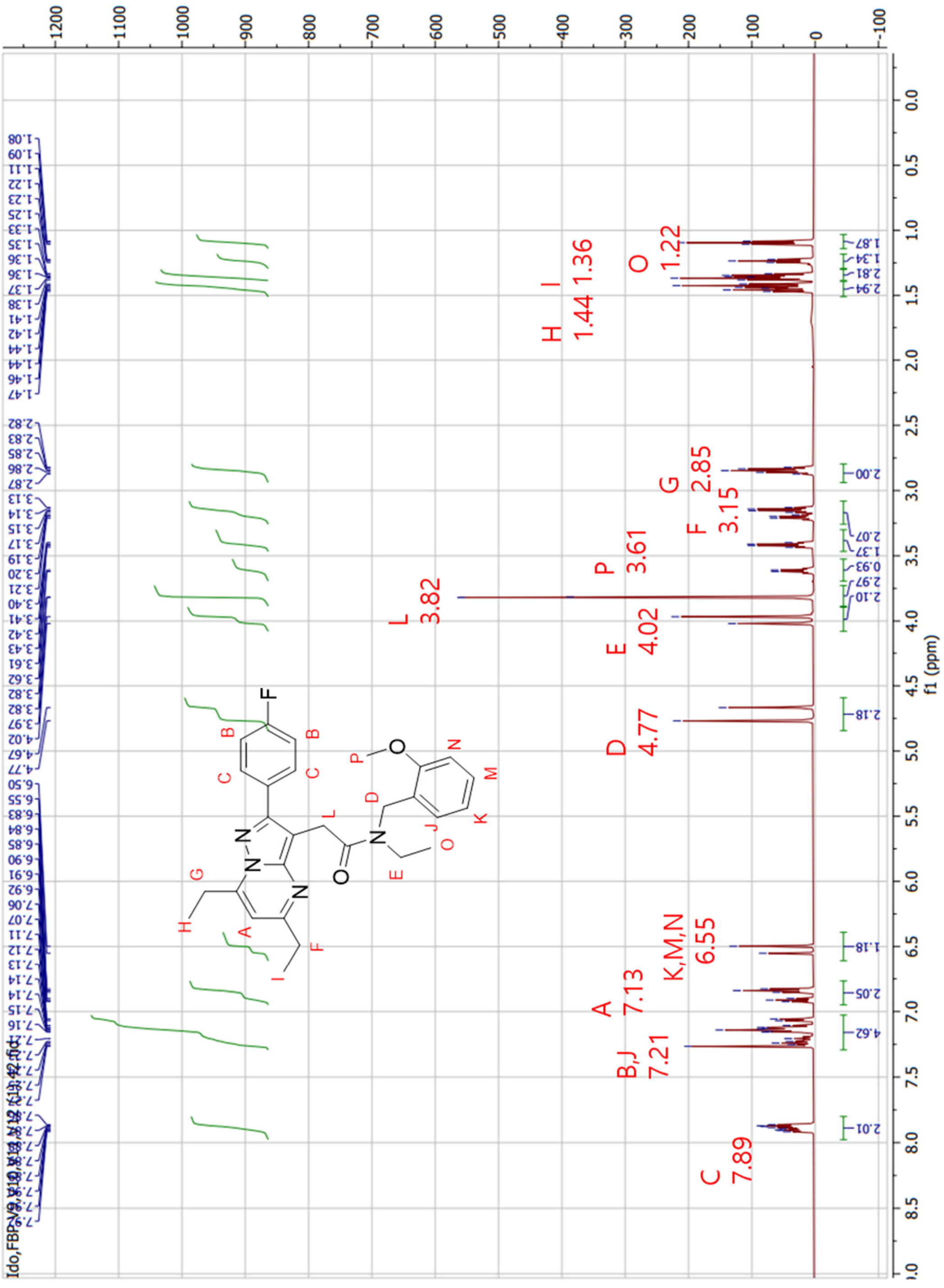

Figure S22. ^{13}C-NMR spectra for **GMA16**

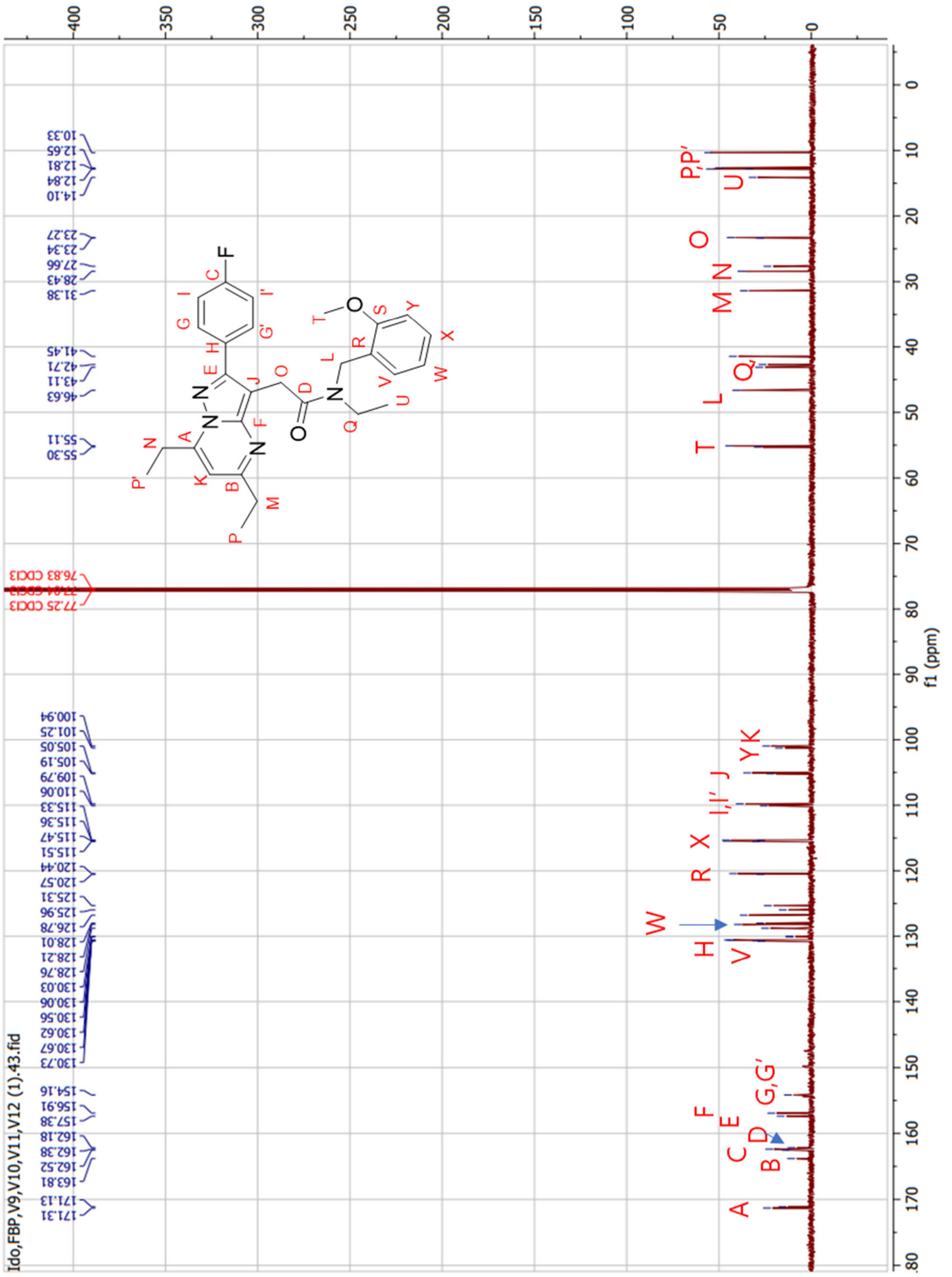

A (171.31), B (163.81), C (162.38), D (hidden), E (157.38) ,F (156.91), GG’ (154.16), H (130.62), II’ (115.51), J (105.19), K (100.94), L (46.63), M (31.38), N (28.43), O (23.34), PP’ (12.95), Q (43.11), R (120.57), S (hidden), T (55.30), U (14.10), V (130.73), W (128.76), X (115.51), Y (101.25)

Figure S23. ^{1}H-NMR spectra for **GMA17**

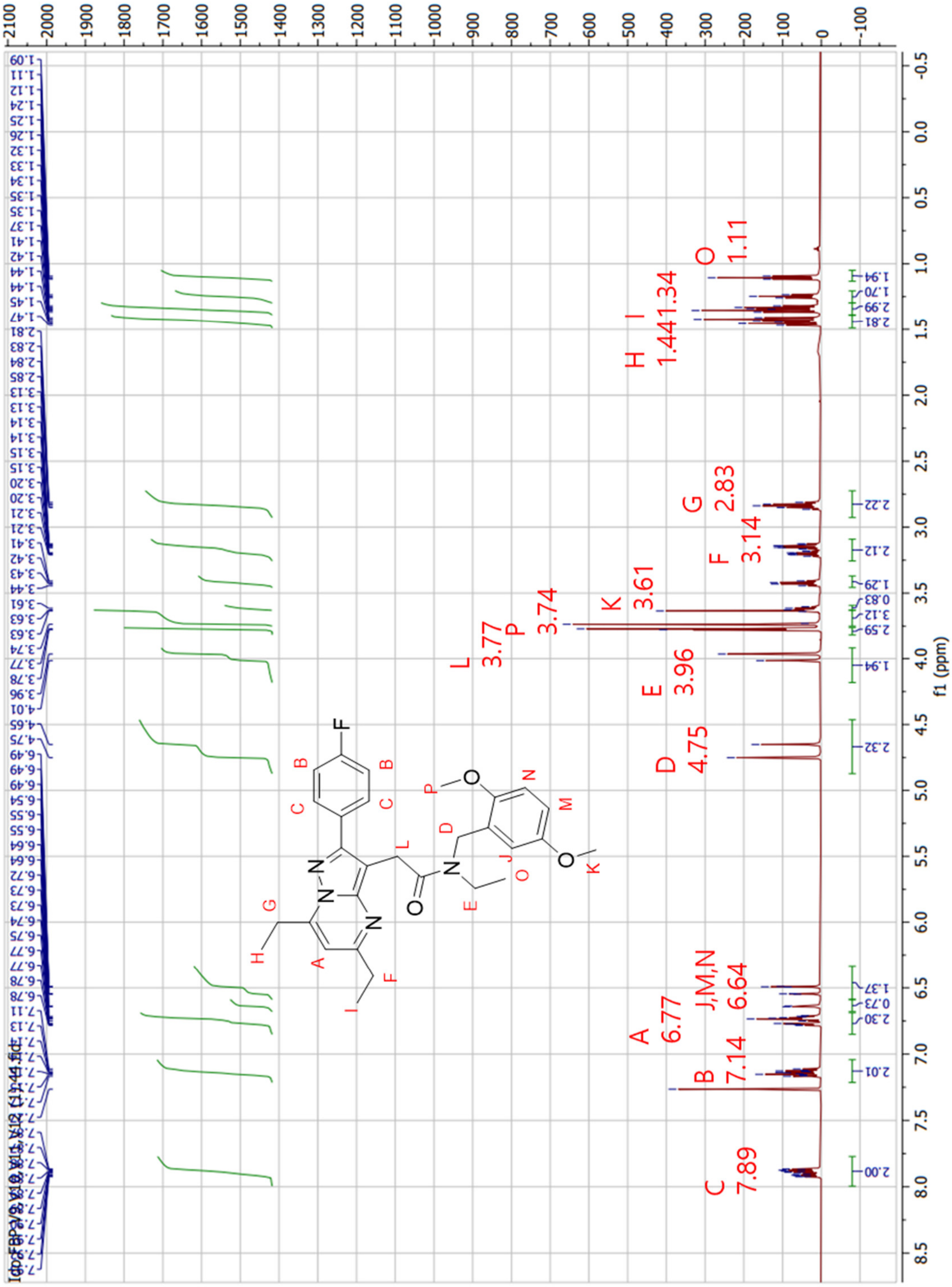

Figure S24. ^{13}C-NMR spectra for **GMA17**

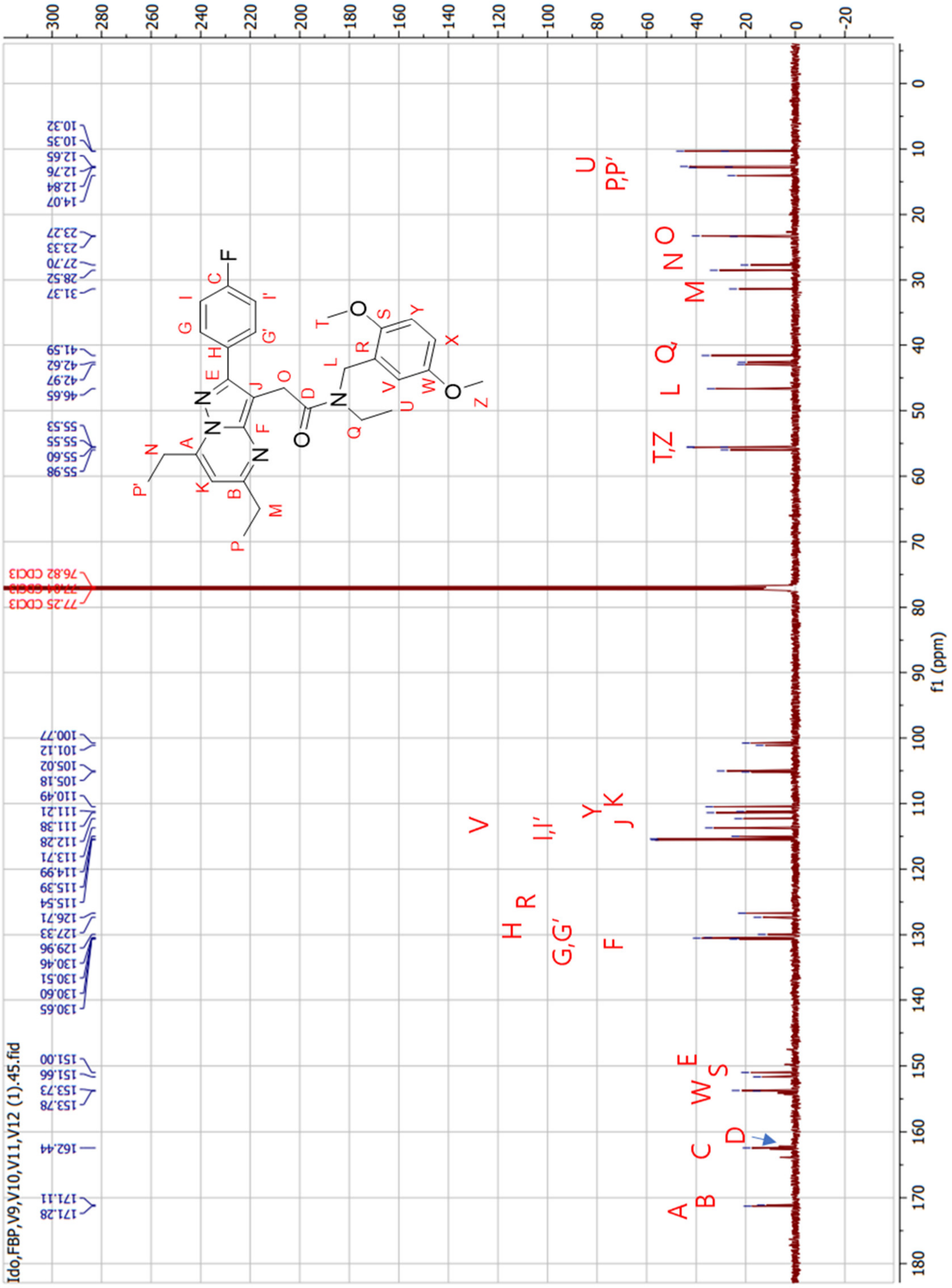

A (171.28), B (171.11), C (162.44), D (hidden), E (151.00) ,F (130.65), GG’ (130.60, 130.51), H (129.96), II’ (115.54, 115.39), J (114.99), K (111.21), L (46.65), M (31.37), N (28.52), O (23.27), PP’ (12.76, 12.84), Q (41.59), R (126.71), S (151.66), T (55.60), U (12.65), V (113.71), W (153.73), X (112.28), Y (111.38), Z (55.55)
